# Univariate-guided sparse regression for Biobank-scale high-dimensional omics data

**DOI:** 10.1371/journal.pgen.1012314

**Published:** 2026-09-23

**Authors:** Joshua Richland, Tuomo Kiiskinen, William Wang, Wenhui Sophia Lu, Balasubramanian Narasimhan, Trevor Hastie, Manuel Rivas, Robert Tibshirani

**Affiliations:** 1 Department of Statistics, Stanford University, Stanford, California, United States of America; 2 Department of Biomedical Data Science, Stanford University, Stanford, California, United States of America; 3 Department of Computer Science, Stanford University, Stanford, California, United States of America; Emory University School of Medicine, UNITED STATES OF AMERICA

## Abstract

We present a scalable framework for computing polygenic risk scores (PRS) in high-dimensional genomic settings using the recently introduced Univariate-Guided Sparse Regression (uniLasso). UniLasso is a two-stage penalized regression procedure that leverages univariate coefficients and magnitudes to stabilize feature selection and produce sparse predictive models. Building on its theoretical and empirical advantages, we adapt uniLasso for application to the UK Biobank, a population-based repository comprising over one million genetic variants measured on hundreds of thousands of individuals from the United Kingdom. We further extend the framework to incorporate external summary statistics via uniLasso ES (external scores). These signals guide the regression toward variants with prior evidence of association by informing penalty weights and sign constraints. Both uniLasso ES and uniLasso ultimately fit multivariate models using individual-level target data; the external statistics guide, rather than replace, this fitting. Our results demonstrate that uniLasso attains predictive performance comparable to standard Lasso while selecting substantially fewer variants, yielding sparser and potentially more interpretable models. Moreover, it remains competitive with other PRS estimation methods, such as PRS-CS and lassosum2.

## Author summary

The growing ability to measure genetic variation across hundreds of thousands of individuals has created new opportunities to investigate the genetic basis of human traits and disease. One common goal is to construct polygenic risk scores, which combine information across genetic variants to predict an individual’s genetic predisposition to a phenotype or disease. However, widely used statistical methods often identify large numbers of variants, making the resulting models difficult to understand. We implement uniLasso, a sparse regression technique that produces simple genetic prediction models while maintaining strong predictive accuracy. The method uses signals from univariate analyses to guide a multivariate regression model toward a smaller set of variants. We further demonstrate how summary statistics from external genetic studies can be incorporated into the uniLasso framework (through uniLasso with external scores, or uniLasso ES), allowing the model to prioritize variants that have previously shown evidence of association. Applying these methods to large-scale genotype data from the UK Biobank, we find that uniLasso (and its extension with external scores) produces substantially sparser models while achieving predictive performance comparable to widely used approaches. These results suggest that combining simple genetic signals and external information with high-dimensional regression can reduce the complexity and improve the accuracy of polygenic risk prediction.

## Introduction

With rapid advancements in computing power and storage capacity, data collection has become increasingly detailed and large in scale. As a result, scientists routinely encounter high-dimensional data settings, where the number of predictors is much larger than the number of observations (p≫n). Such scenarios are common in fields like genomics, where granular information can be recorded for each individual. In these regimes, regression has become a central tool for both prediction and variable selection, underpinning a wide range of modern applications.

Another defining feature of -omics model matrices is the strong correlation among predictors. In genomics, linkage disequilibrium (LD) creates blocks of single nucleotide polymorphisms (SNPs) that carry very similar information; in transcriptomics, co-expression and shared regulatory programs induce clusters of correlated genes. Any multivariate method must decide what to do with these groups: either select a sparse set of representatives, or distribute (“smear”) the effect across many correlated variables.

Often, only a much smaller subset of features have a meaningful relationship with the response. Thus a main goal of researchers is to focus on identifying which variables are worth the investment of time and resources to measure and analyze.

### Review of the Lasso

The Lasso [1] performs both estimation and variable selection and has become a cornerstone of modern statistics and machine learning.

For a model matrix $X\in {\mathbb{R}}^{n\times p}$ and response vector $y\in {\mathbb{R}}^{n}$, Lasso solves the following minimization problem:


$$\begin{array}{c} \hat{\beta }=\underset{\beta }{\text{argmin}}\;\frac{1}{2n}\|y-X\beta {\|}_{2}^{2}+\lambda \|\beta {\|}_{1}. \end{array}$$ (1)

λ is a regularization parameter that determines the number of features identified in the solution set. In practice, we evaluate the objective over a grid of λ values and then select the best λ using either cross-validation or performance on a held-out validation set. Equivalently, we can formulate the Lasso problem in the following way:


$$\begin{array}{c} \hat{\beta }=\underset{\beta }{\text{argmin}}\;\frac{1}{2n}\|y-X\beta {\|}_{2}^{2}\;\text{subject to}\;\|\beta {\|}_{1}\leq t \end{array}$$ (2)

where *t* > 0 is a tuning parameter controlling the sparsity of the solution, directly related to λ in the Lagrangian formulation. The constraint sets an upper bound on how large the coefficients $\hat{\beta }$ can be in total (in terms of their $\ell_{1}$ norm, which is $\sum_{j=1}^{p}|\beta_{j}|$). A larger *t* allows the coefficients to grow by imposing a smaller amount of regularization.

### Optimization approaches for the Lasso

A variety of computational tools have been developed to efficiently solve Lasso problems. One of the most widely cited R packages, glmnet [2], is capable of computing entire solution paths for a range of generalized linear models. Another R package designed specifically for large-scale, high-dimensional Lasso regression in genomics applications is snpnet [3]. In fact, snpnet adapts glmnet and stands out as one of the most competitive solvers, combining strong-rule screening with efficient KKT batching to scale to millions of SNPs. This approach yields high predictive accuracy in large-scale genomic analyses.

Elastic Net (EN) [4] is another path solver that extends the Lasso framework by incorporating an $\ell_{2}$ penalty, thereby combining sparsity with ridge regularization. This generalization provides greater flexibility and stability in settings with correlated features, where pure $\ell_{1}$ regularization may perform suboptimally. EN seeks to minimize the following objective function:


$$\begin{array}{c} \hat{\beta }=\underset{\beta }{\text{argmin}}\left\{\frac{1}{2n}\|y-X\beta {\|}_{2}^{2}+\lambda (\alpha |\beta {|}_{1}+(1-\alpha )|\beta {|}_{2}^{2})\right\}. \end{array}$$ (3)

In equation (3), λ>0 controls the overall regularization strength, while α∈[0,1] balances the contribution of the $\ell_{1}$ penalty and the $\ell_{2}$ penalty. Setting α=1 recovers the Lasso, while α=0 yields ridge regression. This unified structure allows practitioners to interpolate between the two classical approaches within a single flexible framework.

Adelie [5] is a novel Python package (with a version in R as well) designed for efficiently solving Lasso and group Lasso problems. This package can handle large-scale inputs with greater computational efficiency than traditional solvers, and the package provides a suite of generalized linear model (GLM) classes. Benchmarks against other state-of-the-art solvers demonstrate that Adelie achieves faster speeds than other group Lasso packages with equivalent accuracy. On Lasso and EN problems, it performs comparably to the glmnet R package. Additionally, Adelie offers flexible parameter tuning, warm-start capabilities, and easy integration with standard Python data science workflows, making it powerful for high-dimensional regression tasks.

Another notable feature of Adelie, similar to snpnet, is its ability to efficiently fit Lasso and EN models on genome-wide association studies (GWAS). To handle the scale and complexity of such data, Adelie introduces specialized file formats optimized for both memory efficiency and the speed of matrix operations. This design allows the package to process large-scale genotype and phenotype matrices that would otherwise exceed standard memory limits, enabling scalable analyses of GWAS data.

### The UK Biobank database

The UK Biobank [6] provides an excellent testbed for scientists using such regression techniques to investigate quantities of interest, such as the variance explained by genomic predictors, and to identify SNPs potentially associated with specific traits. The UK Biobank is a large, population-based repository containing genetic and health information from approximately 500,000 individuals across the United Kingdom. For each participant, it provides data on over 1,000,000 SNPs alongside a wide range of phenotypes.

Regression provides a natural framework for studying the UK Biobank data, allowing researchers to quantify relationships between SNPs and phenotypes. Packages such as Adelie and snpnet enable analyses to be performed efficiently on such large-scale datasets. By incorporating regularization techniques, i.e., Lasso, regression can produce sparse solutions, effectively reducing the set of over a million SNPs to a smaller subset most likely to be associated with the response. This can not only facilitate interpretation by reducing model complexity, but also improves predictive performance by mitigating overfitting in high-dimensional settings.

Polygenic risk scores (PRS) summarize genetic liability as a weighted sum of genome-wide variants. At Biobank scale, the standard PRS workflow is two-steps: first, perform a univariate GWAS to obtain marginal effect estimates for each SNP; second, apply an LD-aware shrinkage or selection method (for example, clumping and thresholding) to convert these marginal effects into a genome-wide predictor. Historically, even this first step already pushed computational limits (fitting millions of univariate regressions on hundreds of thousands of individuals) so genome-wide multivariate regressions were not practically feasible at scale. This computational constraint, together with the complexity of modelling LD explicitly, is a major reason why univariate GWAS has remained the dominant analysis framework for so long. Because GWAS treats each SNP in isolation, a true signal within an LD block typically lifts an entire neighborhood of correlated SNPs, and without strong shrinkage the naive sum of GWAS betas would imply more variance explained than is empirically possible. The second step exists precisely to correct for this double-counting and sampling noise, but it usually leads to dense predictors with highly flattened per-SNP effect sizes.

### Contributions

In this paper, we implement a novel method for analyzing ultra-high-dimensional -omics data that directly fits a multivariate model on individual-level data, rather than relying on the standard two-step GWAS-plus-PRS pipeline. We present Univariate-Guided Sparse Regression (uniLasso) [7] tailored for biobank-scale SNP data. Empirically, uniLasso achieves predictive performance comparable to existing methods but with approximately 40% fewer predictors, resulting in a substantially more parsimonious model.

Additionally, we adapt uniLasso to integrate external data sources to further enhance predictive accuracy. In biomedical research, privacy regulations often prevent the sharing of raw data, so researchers sometimes may only provide summary statistics rather than entire data sets. Following the approach proposed in [7], we demonstrate how to incorporate external scores from independent datasets into the uniLasso pipeline. This alternative approach to uniLasso uses external summary statistics to guide penalty weights and/or sign constraints, rather than replace model fitting. We combine these external scores with individual-level data in a single sparse multivariate model, rather than as a separate post-GWAS step. Prior hybrid approaches instead combine information at the level of GWAS results or final scores (e.g., meta-PRS [8]), integrating external information only after model fitting rather than within the regression itself. This also distinguishes our approach from strictly summary-statistics-based methods.

This paper is organized as follows: The next section introduces our adaptation of the uniLasso algorithm, beginning with a brief recap of the standard method before describing how we extend it to both continuous and binary response variables in ultra-high-dimensions. We outline precisely how to integrate external scores in this section as well. In the following section, we demonstrate the practical use of uniLasso through an application to the UK Biobank. We present additional experiments highlighting the benefits of incorporating external data from an independent source and summarize comparisons of uniLasso to other approaches for constructing polygenic risk scores. We conclude by outlining future challenges and discussions. Finally, while the work in this paper is tailored to genomic prediction, the methodology and steps we describe extend naturally to non-genomic settings.

## Materials and methods

### Overview of the uniLasso algorithm

Univariate-Guided Sparse Regression (uniLasso) [7] is a two-stage regression framework that promotes sparsity and is designed to enhance interpretability by leveraging information from univariate regressions. Specifically, uniLasso constrains the coefficients in a multivariate model to align with the signs of their corresponding univariate coefficients, enhancing predictive accuracy and potentially facilitating interpretation of selected predictors.

The uniLasso procedure, as outlined in [7], assumes the same supervised learning framework as Lasso. For a model matrix $X\in {\mathbb{R}}^{n\times p}$ and response vector $y\in {\mathbb{R}}^{n}$, the uniLasso procedure is defined as follows:

1 For j=1,2,...,p compute the univariate predictions $\hat{\eta_{ij}}={\hat{\beta }}_{0j}+{\hat{\beta }}_{j}x_{ij}$ as well as their leave-one-out (LOO) counterparts

$$\begin{array}{c} {\hat{\eta }}_{ij}^{-i}\;=\;{\hat{\beta }}_{0j}^{-i}+{\hat{\beta }}_{j}^{-i}x_{ij},\;i=1,...,n,\;j=1,...,p. \end{array}$$

2 Fit a non-negative Lasso with an intercept and no standardization to target *y* using the LOO univariate predictions as features:

$$\begin{array}{cc} & \underset{\theta }{\text{argmin}}\;\left\{\frac{1}{n}\sum_{i=1}^{n}(y_{i}-\theta_{0}-\sum_{j=1}^{p}{\hat{\eta }}_{ij}^{-i}\;\theta_{j}{)}^{2}+\lambda \sum_{j=1}^{p}\theta_{j}\right\}\\ & \text{subject to}\;\theta_{j}\geq 0,\;j=1,...,p.\text{ Select }\lambda \text{ through cross validation.} \end{array}$$

3 The final model can be written as

$$\begin{array}{c} \hat{\eta }(x)={\hat{\gamma }}_{0}+\sum_{j=1}^{p}{\hat{\gamma }}_{j}x_{j}, \end{array}$$

with ${\hat{\gamma }}_{j}={\hat{\beta }}_{j}{\hat{\theta }}_{j}$, and ${\hat{\gamma }}_{0}={\hat{\theta }}_{0}+\sum_{\ell =1}^{p}{\hat{\beta }}_{0\ell }{\hat{\theta }}_{\ell }$.

Note that we do not standardize the features before applying the non-negative Lasso in Step 2 because the values ${\hat{\eta }}_{ij}^{-i}$ are all on the scale of the response. Closed-form expressions for the LOO fits and the univariate coefficients are provided later in this section.

The steps taken in this procedure serve several purposes. First, it ensures that the signs of the final coefficients agree with the signs of the univariate coefficients (or they are zero). This increases the possible interpretability of the result, as it excludes features whose coefficient sign changes between the univariate and multivariate model. In addition, features with larger univariate coefficients will also tend to have larger coefficients in the final model, since they are given an advantage in the penalization of θ. Moreover, using LOO fitted values rather than raw univariate estimates or the original features amounts to feeding cross-validated univariate predictions into the Lasso in Step 2. This forces the selection step to rely on out-of-sample predictive signal rather than in-sample noise, which empirically leads to sparser models and improved generalization [7]. It also justifies the use of cross validation.

As demonstrated in the uniLasso paper [7], this method has exhibited excellent performance, producing sparser solutions than classical techniques. In its original formulation, however, uniLasso is not immediately scalable to biobank-sized datasets, which prevents a direct comparison with genomics tools such as snpnet. In what follows we will discuss some of the challenges that emerge when applying the algorithm in the high dimensional regime.

One challenge posed by uniLasso in ultra-high-dimensional data analysis is the need to store the immense feature matrix. In computation, Step 1 of the uniLasso pipeline actually requires the user to store an additional (n×p) matrix of the LOO fitted values. While SNP matrices are typically sparse (many of the entries are 0 and all can be stored in at most 2 bits), the LOO matrix is not. Thus the computer must be capable of storing another feature matrix which could take up around 1500 GB of memory (see S2 Appendix for a more in depth discussion of memory usage). We recognize that this storage requirement may present a computational bottleneck for applying uniLasso at this scale. Later in the manuscript, we introduce uniLasso ES, a related formulation that leverages external summary statistics and therefore does not require construction of the full LOO matrix, substantially reducing memory requirements.

Computation arises as a second challenge when calculating the univariate coefficients and LOO fitted values. These need to be computed for each column, when the number of covariates is in the order of millions (as is the case in -omics settings).

A final challenge arises in Step 2 of the algorithm when choosing the regularization value. Again due to the scale of the data, cross validation becomes computationally infeasible. This is because each of the *k* folds requires refitting the model on a large proportion of the entire dataset, leading to prohibitively large computational, memory, and time costs.

### Details of the omics-adapted uniLasso algorithm

To address the computational challenges discussed above, we propose a modified version of the uniLasso algorithm designed for application in general -omics settings. We demonstrate that this method achieves predictive performance comparable to standard tools such as snpnet, while producing substantially sparser models with reduced complexity.

We use Sherlock, a high-performance computing (HPC) cluster at Stanford University, to engineer the uniLasso pipeline using the Adelie Python package. Our compute instances use a cluster of Intel(R) Xeon(R) E5-2640 v4 CPUs with 64 total cores and ~3TB RAM. Methods were run using 64 threads. This setup enables efficient allocation of memory and computational resources necessary to sustain the full analysis. When calculating the leave-one-out (LOO) fits and univariate coefficients, it is important to note that each computation depends only on individual columns of the design matrix.

For a given SNP column *x*, let $\tilde{x}=x-\bar{x}$, where $\bar{x}$ denotes the column mean. Similarly, define *y* as the response and $\tilde{y}=y-\bar{y}$ as its centered counterpart. The univariate slope *u*_1_ and intercept *u*_0_ are then given by:


$$\begin{array}{c} u_{1}=\frac{{\tilde{x}}^{⊤}\tilde{y}}{\|\tilde{x}{\|}^{2}},\;u_{0}=\bar{y}-u_{1}\cdot \bar{x}. \end{array}$$

The vector of LOO fitted values *f* corresponding to the column *x* are defined as follows, where $H_{ii}$ denotes the $i^{th}$ diagonal element of the hat matrix *H*:


$$\begin{array}{c} y_{i}-f_{i}=\frac{y_{i}-(u_{0}+u_{1}x_{i})}{1-H_{ii}},\;\text{where }H_{ii}=\frac{1}{n}+\frac{{\tilde{x_{i}}}^{2}}{\|\tilde{x}{\|}^{2}}. \end{array}$$

Using vectorized formulas suggested by [7], we efficiently implemented computations that are embarrassingly parallel, allowing us to fully exploit CPU resources and markedly accelerate computation.

In the standard uniLasso algorithm, the regularization parameter in Step 2 is typically chosen via *k*-fold cross validation. However, once again given the scale of our data, this is computationally infeasible. Instead, following the approach of [3], we employed a train-validation-test split (70/10/20) to efficiently tune the regularization strength. Specifically, we first fit the model on the training set over a grid of λ values. Using the validation set, we then identified the value of λ that achieved the best predictive performance. Next, we refit the model on the combined training and validation sets with the chosen parameter. Finally, we evaluated the resulting model on the held-out test set to estimate its out-of-sample performance.

This validation-based approach substantially reduced computational time and the need to store large amounts of data, while yielding empirically strong results. We also believe that this strategy is an effective and generalizable approach for hyperparameter tuning in ultra-high-dimensional settings.

Moreover, SNP data is known to be particularly sparse, typically taking values in the set {0, 1, 2}. Oftentimes, a majority of the entries in a given column of a SNP are 0. To address the small levels of variance within certain columns, we implemented two strategies:

1 **Minor allele frequency (MAF) filtering:**

MAF is the proportion of the less common allele among all observed alleles at each SNP. SNPs with MAF below 0.0005 were excluded prior to applying uniLasso (or Lasso). We intentionally use a more permissive threshold to retain low-frequency variants that may still carry predictive signal at biobank scale. This filtering step is standard in genomic analyses, as rare variants tend to contribute little predictive power and can lead to unstable coefficient estimates.

2 **Stabilizing regularization of low-variance columns:**

For SNPs with MAF ≥0.0005, small variances still occasionally caused univariate coefficients to explode, leading to unstable estimates. One key quantity that appears in the denominator of the univariate and LOO calculations is


$$\begin{array}{c} S_{xx}={\tilde{x}}^{⊤}\tilde{x},\;\text{where }\tilde{x}=x-\bar{x}. \end{array}$$

[9] encountered a similar instability in the context of microarray analysis, where small denominators led to highly variable test statistics. They propose adding a data-dependent constant to stabilize the denominator. Following this approach, we regularize SNP columns by replacing $S_{xx}$ with


$$\begin{array}{c} S_{xx}^{*}=S_{xx}+\text{fifth-percentile}(S_{xx}), \end{array}$$

where the additive constant corresponds to the fifth percentile of the empirical distribution of $S_{xx}$ values over all columns of the SNP matrix. This adjustment prevents unstable estimates arising from sparse or near-constant genotype columns when $S_{xx}$ approaches zero.

Both modifications substantially improved stability across data partitions and, in most cases, even enhanced predictive performance relative to non-regularized runs. These adjustments may be unnecessary when applying uniLasso in non-genomic settings.

### Omics-adapted uniLasso algorithm for binary response variables

In the case where the response variable is binary rather than continuous ($y_{i}\in \{0,1\}$ for all *i*), we use a penalized logistic regression objective function:


$$\begin{array}{cc} \hat{\beta }= & \underset{\beta }{\text{argmin}}\left\{-\frac{1}{n}\sum_{i=1}^{n}\left[y_{i}\log p_{i}+(1-y_{i})\log(1-p_{i})\right]+\lambda \sum_{j=1}^{p}|\beta_{j}|\right\},\\ & \text{where }p_{i}=\frac{\exp(\beta_{0}+x_{i}^{⊤}\beta )}{1+\exp(\beta_{0}+x_{i}^{⊤}\beta )}. \end{array}$$

Similar to the Lasso, we introduce regularization to control the sparsity of the solution.

For uniLasso, recall that the algorithm requires LOO fitted values and univariate coefficients. Unlike the case with Gaussian loss, there are no closed-form solutions for these expressions. However, in practice, logistic regression is typically fit via iteratively reweighted least squares (IRLS). We follow the procedure described in [7], summarized below:

Given the fitted linear predictor vector $\eta_{j}^{\left(\ell \right)}$ at iteration ℓ, one forms a working response vector $z_{j}^{\left(\ell \right)}$ that depends on $\eta_{j}^{\left(\ell \right)}$, *y*, and the GLM family, along with an observation weight vector $w_{j}^{\left(\ell \right)}$. The updated model $\eta_{j}^{\left(\ell +1\right)}$ is then obtained by performing weighted least squares of $z_{j}^{\left(\ell \right)}$ on $x_{j}$ with weights $w_{j}^{\left(\ell \right)}$. In our implementation, we perform two IRLS iterations per feature.

As in the Gaussian setting, we parallelize and vectorize operations to efficiently fit all *p* univariate logistic regression models. The expressions are only slightly more complex than in the unweighted case. For the LOO fits, we adopt the approximation proposed by [10], which provides estimated LOO predictions without refitting and has been shown to work well in large-scale GLM applications. This corresponds to using the final weighted least squares IRLS iteration from each univariate fit when fitting the univariate models. We use the formula they propose which accommodates observation weights.

Uniquely, we apply the same regularization and MAF filtering when running the binary uniLasso algorithm. Aside from the modifications required for the logistic loss and the corresponding univariate values, the majority of the uniLasso pipeline remains nearly identical to the Gaussian case.

### UniLasso with external scores

A collaborator from Finland provided external summary statistics from FinnGen [11], the Finnish counterpart to the UK Biobank, which comprises approximately 500,000 unique participants. The external scores from FinnGen, denoted E, consist of the results of univariate SNP regressions that include age, sex, and the first ten principal components as covariates.

Rather than relying on the standard formulation that uses leave-one-out (LOO) coefficients [7], propose an alternative regression framework, presented as equation (3.3) in their paper. Importantly, the final model is still fit on individual-level data, with external statistics used only for guidance:


$$\begin{array}{c} \underset{\gamma }{\text{argmin}}\sum_{i=1}^{n}(y_{i}-\gamma_{0}-\sum_{j=1}^{p}x_{ij}\gamma_{j}{)}^{2}+\lambda \sum_{j=1}^{p}\frac{\left|\gamma_{j}\right|}{\left|{\tilde{\beta }}_{j}\right|}\;\text{ s.t. sign}(\gamma_{j})=\text{sign}({\tilde{\beta }}_{j})\;\forall j \end{array}$$ (4)

The idea is to use the univariate coefficients from E rather than compute the LOO estimates from the training data. In equation (4), these correspond to the ${\tilde{\beta }}_{j}$ terms. Specifically, the magnitudes $\left|{\tilde{\beta }}_{j}\right|$ serve as adaptive weights in the $\ell_{1}$-penalty term, while the signs of ${\tilde{\beta }}_{j}$ agree with the signs of the estimated coefficients $\gamma_{j}$ (or they are zero). We draw the univariate slopes and intercepts from the UK Biobank training data, but use the external coefficients ${\tilde{\beta }}_{j}$ to define the adaptive penalty weights and sign constraints in the second-stage regression. The final model can be written as $\hat{\eta }(x)={\hat{\gamma }}_{0}+\sum_{j=1}^{p}{\hat{\gamma }}_{j}x_{j}.$ Note this alternative construction holds for binary responses as well.

## Results

### UK Biobank analysis

We describe a real-data application on the UK Biobank that demonstrates the utility of the uniLasso algorithm.

The UK Biobank [6] is a large prospective cohort study containing extensive genetic and phenotypic data from approximately 500,000 middle-aged individuals across the United Kingdom. In this analysis, we focus on modeling the relationship between individual genotype sequences and their corresponding phenotypic traits. Using uniLasso, we aim to identify SNPs that are most informative for predicting the phenotype of interest. Our proposed adaptation enables model fitting at full scale and yields a reduced active variable set compared to alternative methods. This smaller collection of selected SNPs maintains comparable levels of variance explained.

We restricted our analysis to a subset of 336,442 White British individuals from the full UK Biobank dataset who satisfy the population stratification criteria described in [12]. The data were randomly partitioned into training (70%), validation (10%), and test (20%) subsets. Each individual has 1,080,968 measured genetic variants, where each variant is encoded by one of three levels: 0 for homozygous major alleles, 1 for heterozygous alleles, and 2 for homozygous minor alleles. This set reflects a quality-controlled collection of UK Biobank variants consisting primarily of directly genotyped SNPs, together with CNV (copy number variation) calls and HLA (human leukocyte antigen) alleles derived from array data, after standard filtering procedures based on missingness, minor allele frequency, and Hardy-Weinberg equilibrium [13]. Missing genotypes were imputed using the mean of the observed values.

In addition to genotype data, covariates such as age, sex, and 10 precomputed principal components of the SNP matrix were included to account for population structure. See S3 Table for the predictive performance of the covariate-only model (age, sex, and principal components), which serves as a baseline for evaluating the incremental predictive contribution of genetic predictors reported in S10 Table. Among the thousands of measured phenotypes in this dataset, the phenotypes we used to evaluate our method were standing height, body mass index (BMI), coronary heart disease (CHD), and asthma. CHD and asthma are binary responses, where 1 indicates disease and 0 no disease.

To evaluate predictive performance for the continuous responses, we use the *R*^2^ statistic: given a linear estimator $\hat{\beta }$ and data (*y*, *X*), *R*^2^ is defined as


$$\begin{array}{c} R^{2}=1-\frac{\|y-X\hat{\beta }{\|}_{2}^{2}}{\|y-{\bar{y}}_{0}\text{1}{\|}_{2}^{2}}, \end{array}$$

where ${\bar{y}}_{0}$ is the average response of the training data and **1** is the *n*-vector of all ones. The PRS for individual *i* is the linear predictor ${\hat{PRS}}_{i}=x_{i}^{⊤}\hat{\beta }$, where $x_{i}$ denotes that individual’s vector of SNP genotypes. In this formulation, covariates enter only through the evaluation model used to predict the phenotype itself. The resulting *R*^2^ quantifies the proportion of variance explained by this combined model. Ranging from 0 to 1, higher values represent better explanatory power by the model.

For binary responses, we assess predictive performance using the receiver operating characteristic (ROC) curve, which illustrates the trade-off between true and false positive rates across different classification thresholds. The area under the ROC curve (AUC) reflects how well the model distinguishes between positive and negative outcomes, with values ranging from 0.5 (no discriminatory ability) to 1 (perfect discrimination). For phenotypes with binary outcomes, we evaluate the AUC for all regression methods used.

We perform Elastic Net (EN), Lasso, and uniLasso on all of the aforementioned phenotypes to have a sense of any potential advantages. As in [3], we fit the elastic-net along separate solution paths over λ for α∈{0.1,0.5,0.9}, and evaluate performance (*R*^2^ or AUC) on the validation set. We then select the best (α,λ) pair and report the corresponding test performance. For height, EN selects α=0.9 based on validation performance, while for CHD, BMI, and asthma it selects α=0.1.

Fig 1 illustrates the difference in their performance on held out test sets while Table 1 displays the number of non-zero coefficients at the optimal λ value for each method and phenotype. To quantify uncertainty, we include standard error estimates for all performance metrics. The standard error of *R*^2^ is derived via the delta method, while the standard error of the AUC can be estimated and upper bounded by 1/(4min(m,n)), where *m* and *n* denote the number of cases and control observations [3,14,15]. For height and BMI, these standard errors are approximately 0.002 and 0.007, respectively, and for asthma and CHD, they are upper bounded by 0.005 and 0.009, respectively. Because these standard errors are driven primarily by phenotype prevalence and test-set size, they vary negligibly across methods within a phenotype. We therefore compute the delta-method standard errors using the uniLasso predictions and use the resulting estimates for all methods within the corresponding phenotype. These estimates are reflected as error bars in Fig 1.

**Table 1:** Number of non-zero coefficients for each phenotype measured across all Lasso-based algorithms. Compared to Lasso, uniLasso selects 38% fewer SNPs for height and 43% fewer for BMI, while only reducing *R*^2^ by 0.006 and 0.016, respectively.

Number of Non-Zero Coefficients				
Model	Height	BMI	CHD	Asthma
Lasso	55,038	33,076	2,615	5,556
Elastic Net	55,230	43,987	5112	7390
uniLasso	**34,256**	**18,833**	**1,009**	**3,030**
uniLasso ES	44,788	24,598	1,310	5,698

**Fig 1:**
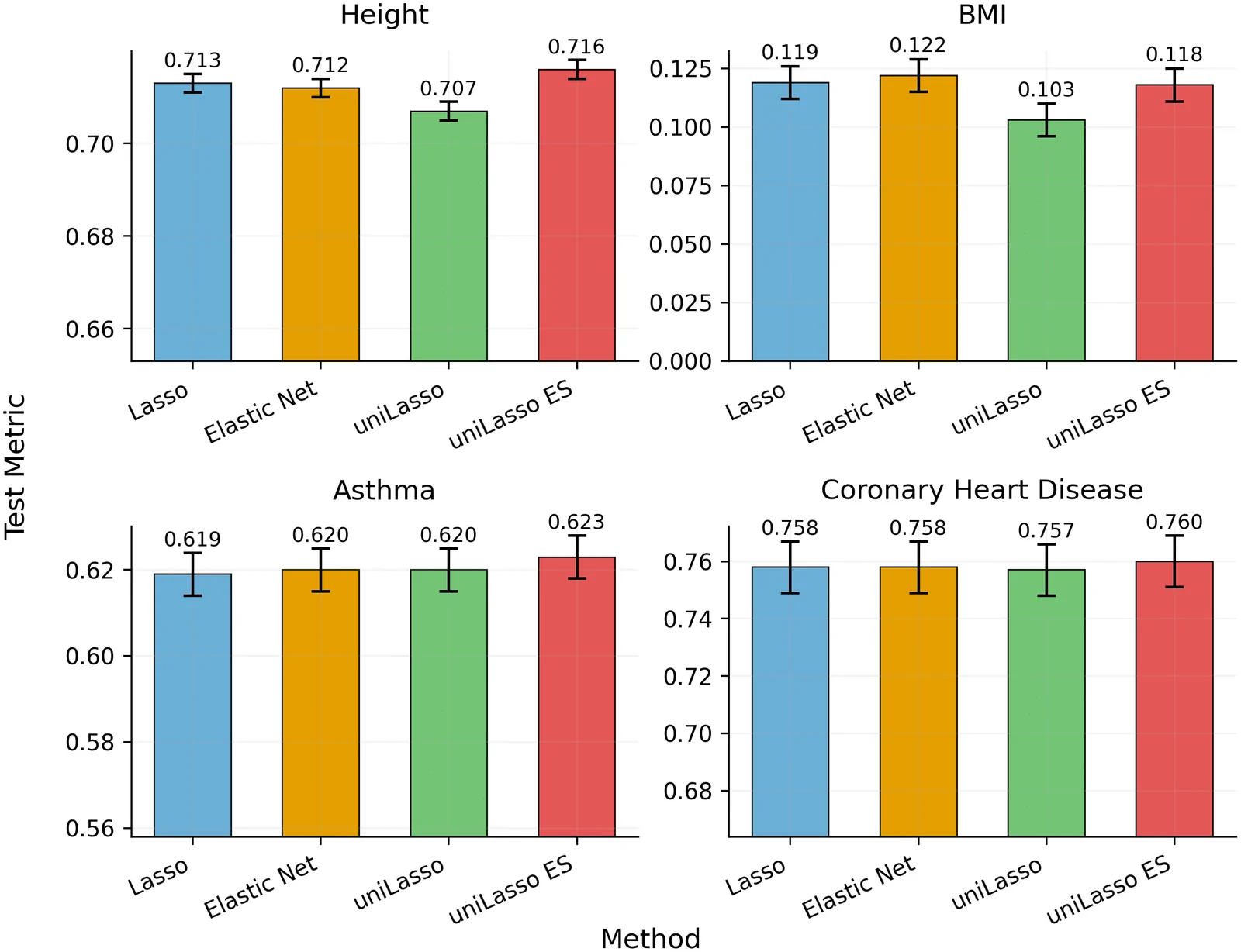
Prediction Comparisons for Lasso-based methods. Comparison of the test set predictive performance of the different polygenic risk score (PRS) methods with refitting on the training and the validation set. All regressions were performed with Adelie in Python. Test-set *R*^2^ is the metric evaluated for the continuous phenotypes (Height and BMI), and AUC is evaluated for the binary phenotypes (Asthma and CHD).

For the continuous phenotypes (height and BMI) there is a consistent trend: Lasso achieves higher point estimates than uniLasso by a small margin but uses a significantly larger number of coefficients in its solution. The small loss in *R*^2^ is worthwhile for uniLasso when considering the nearly 40% reduction in active-set size. Elastic Net does slightly improve upon Lasso for BMI but uses a larger support size for both phenotypes.

In the binary case, uniLasso and Lasso perform much more similarly as measured by AUC, with uniLasso slightly outperforming Lasso on asthma, and vice versa on CHD. EN does not meaningfully improve upon Lasso (aside from a marginal 0.001 gain on asthma) and, again, consistently uses more variables. Excitingly, as also seen with the continuous responses, uniLasso maintains a significantly sparser solution set than Lasso (See Table 1). For CHD, uniLasso is over 50% sparser than Lasso and has lower test error by only 0.001. This reduced solution set in conjunction with the alignment of signs between univariate and multivariate coefficients reinforces the merits of uniLasso. See S2 Table for additional evaluation measures (PR-AUC and calibration) for the binary traits, which show similar performance across methods. The next section goes into more detail regarding the incorporation of external summary statistics to potentially achieve a more optimal tradeoff.

Table 2 summarizes the number of sign changes observed between the univariate coefficients and the non-zero coefficients in the final Lasso model. Eliminating such sign changes can be viewed as an additional form of regularization: a sign flip indicates that the multivariate fit has reversed the direction of association for a variable. For ultra-high-dimensional, noisy settings, imposing this extra regularization is well-motivated. As shown in the table, the binary models exhibit far fewer sign changes than the continuous ones, which is expected given their smaller active-sets. A built-in advantage of uniLasso is the lack of sign changes regardless of the type of response variable.

**Table 2:** Lasso sign changes between univariate and multivariate models. This table displays the number of coefficient sign changes between the univariate and multivariate models for each phenotype and the percentage of coefficients in the active-set that switched. UniLasso, by construction, eliminates all such sign reversals; every non-zero coefficient retains the sign of its univariate effect.

Number and Percentage of Sign Changes in Lasso		
Phenotype	# Sign Changes	% Sign Changes
Height	6,698	12%
BMI	103	0.3%
CHD	2	≤ 0.01%
Asthma	2	≤ 0.01%

### Use of external scores

Our second contribution addresses the integration of external data to enhance predictive accuracy. While uniLasso introduces the core modeling framework, uniLasso ES provides a practical extension that leverages external summary statistics to often improve predictive performance and support more efficient modeling in high-dimensional settings. Due to privacy considerations in biomedical research, access to individual-level data is frequently restricted, with researchers often sharing only summary statistics. [7] proposed a method for incorporating external univariate scores from an independent dataset into the uniLasso pipeline, which was discussed in more detail in the previous section.

The results for uniLasso with external scores (denoted uniLasso ES) are displayed alongside those of Lasso and standard uniLasso in Fig 1. UniLasso ES achieves the highest predictive performance across all phenotypes evaluated except for a negligible 0.001 difference with Lasso for BMI. Compared with standard uniLasso, incorporating external scores improved predictive performance across the phenotypes evaluated in this study. Although the resulting models are generally less sparse than standard uniLasso, they remain sparser than Lasso for three of the four phenotypes and are comparable in sparsity for asthma (Table 1). The solution set for CHD is also very nearly 50% sparser than Lasso in this framework. These findings demonstrate that incorporating external scores enhances accuracy while yielding models that exceed the ubiquitous Lasso in predictive power and sparsity.

Table 2 shows that the binary models exhibit very few sign changes. This naturally leads to examining how much alignment there is among the sets of selected SNPs across methods. Table 3 reports the sizes of each pairwise intersection divided by the size of their union. We examine the overlaps between Lasso, uniLasso, and uniLasso ES. Across all phenotypes, Lasso and uniLasso share the largest overlap, followed by Lasso and uniLasso ES. Lasso and uniLasso exhibit their largest similarity for the binary traits. A stability analysis of the uniLasso coefficients is presented in S1 Fig.

**Table 3:** Proportion of common features. This displays the proportion of shared nonzero coefficients between each pair of models (Lasso (L), uniLasso (U), and uniLasso with external scores (ES)) for all phenotypes. Each value represents the size of the intersection divided by the size of the union of non-zero SNPs. Recall that the binary phenotypes have much smaller active-sets, as shown in Table 1.

Similarity	Height	BMI	CHD	Asthma
*L* and *U*	0.27	0.29	0.29	0.36
*L* and *ES*	0.20	0.18	0.07	0.10
*U* and *ES*	0.23	0.22	0.09	0.11

### Comparison of uniLasso to other approaches

In this section we summarize the performance differences among the methods considered with three other summary statistic based approaches. In addition to the four Lasso-based methods, we first analyzed PRS-CS [16], a summary statistics-based Bayesian regression method. PRS-CS computes polygenic risk scores using GWAS summary statistics and an external LD reference panel. Unlike uniLasso ES, however, it operates entirely on summary statistics rather than fitting an individual-level regression model in which summary statistics are used to guide the estimation. Furthermore, PRS-CS shrinks effect-size estimates toward zero but does not produce exact zero coefficients. For PRS-CS, we first generated GWAS summary statistics on the training set using age, sex, and the top 10 PCs as covariates in PLINK v2.0a7, restricting variants to MAF ≥0.01 [17,18]. We then applied PRS-CS using the standard European HapMap3 LD reference panel derived from the 1000 Genomes Project (PRScs GitHub Repo) which included 750,510 variants. To avoid overfitting, the PRS-CS global shrinkage parameter (ϕ) was tuned on the validation set from the candidate values $\left\{auto,10^{-2},10^{-4},10^{-6}\right\}$. For each candidate ϕ, PRS were computed on the validation set. For summary-statistic methods, the PRS enters the prediction model with a regression coefficient that must be estimated, since these methods return scores in arbitrary units. For every method, this coefficient, together with the covariate coefficients, was estimated on data independent of the held-out test set and then held fixed when evaluating test-set performance. After selecting the optimal ϕ, the resulting prediction model was applied unchanged to the held-out test set. No coefficient or hyperparameter was estimated on the test set (see S6 Table for a full breakdown of PRS-CS results).

As described in greater detail in S3 Appendix, we evaluate PRS-CS in three configurations: FinnGen-only, UK Biobank only, and FinnGen-UK Biobank-Meta. Here we report the results from the FinnGen-UK Biobank-Meta configuration, in which FinnGen summary statistics were meta-analyzed with UK Biobank training-only GWAS summary statistics using inverse-variance weighting. The resulting PRS was then combined with covariates, calibrated in the validation cohort, and evaluated in the held-out test cohort. This benchmark is intentionally strong for standard summary-statistic-based PRS methods, as it leverages both external FinnGen information and additional UK Biobank GWAS information before PRS construction. We focus on this configuration because it produced the strongest PRS-CS results and was therefore the most competitive benchmark. It is worth noting that in the UK Biobank-only analyses, PRS-CS did not have higher point estimates than Lasso, EN, or uniLasso ES.

Although both uniLasso ES and the FinnGen-UK Biobank-Meta PRS incorporate external information, they do so in fundamentally different ways. The meta-analysis approach increases GWAS power prior to PRS construction by combining summary statistics across studies. In contrast, uniLasso ES uses external effect estimates to inform penalty weights and sign constraints within an individual-level sparse regression model. As a result, the comparison is informative from a predictive-performance perspective, but the two approaches leverage external information through different statistical mechanisms.

With the meta configuration, though, PRS-CS holds the highest point estimate on two phenotypes, BMI and CHD, by margins of 0.010 and 0.006, respectively, relative to the best-performing Lasso method (see Table 4). Combined with its lack of sparsity, this limits its suitability for settings where a smaller active set is desirable. In particular, the dense set of nonzero effects makes it harder to gain intuition behind which variants might be driving the signal. Moreover, because PRS-CS draws from a larger pool of variants, it has greater capacity to capture polygenic signal.

**Table 4:** Test set predictive performance of five PRS methods for each phenotype. For Height and BMI, the metric is *R*^2^; for asthma and CHD, the metric is AUC. UniLasso with external scores is denoted as uniLasso ES. PRS-CS is a Bayesian polygenic prediction method, while lassosum2 is a summary-statistics-based regression approach.

Phenotype	Lasso	uniLasso	uniLasso ES	PRS-CS	lassosum2
Height	0.713	0.707	**0.716**	0.681	0.675
BMI	0.119	0.103	0.118	**0.129**	0.122
Asthma	0.619	0.620	**0.623**	0.617	0.591
CHD	0.758	0.757	0.760	**0.766**	0.757

We also compare against lassosum2 [19], a summary-statistics-based penalized regression method for PRS construction. This additional Lasso-based method uses GWAS summary statistics together with linkage disequilibrium (LD) information from a reference panel to estimate SNP effect sizes. We run lassosum2 on the same three external summary statistic configurations used for PRS-CS, but once again only present the FinnGen-UK Biobank-Meta configuration because it yielded the best results (Table 4). Overall, lassosum2 performs worse than all of the Lasso-based methods except on BMI, where it has a higher overall point estimate than the best-performing model by 0.003 in *R*^2^. Unlike PRS-CS and other traditional summary-statistics-based methods, lassosum2 promotes *some* sparsity in its coefficients, but not nearly to the extent of any of the Lasso-based methods. S7 Table displays the number of active variants with nonzero lassosum2 weights, while confidence intervals and point estimates for all configurations are shown in S8 Table.

Finally, we evaluated clumping-and-thresholding (C + T) as a matched-sparsity sensitivity analysis, selecting approximately the same number of variants as the corresponding uniLasso score. This simple pruning strategy yielded lower point estimates than uniLasso in seven of eight C + T Union or C + T Meta cells. Relative to standard uniLasso, the differences ranged from −0.060 to +0.002 in *R*^2^/AUC units. Relative to uniLasso ES, the C + T point estimates were lower in all eight cells. Full C + T results are shown in S9 Table.

S3 Appendix contains a lengthier discussion of the implementation details, hyperparameter tuning, and computational considerations for PRS-CS, lassosum2, and C + T.

## Discussion

In this paper, we propose a new framework for analyzing ultra-high-dimensional -omics data. Our approach builds on the univariate-guided sparse regression (uniLasso) algorithm recently introduced by [7]. More broadly, our work demonstrates how uniLasso can be adapted into a practical framework for biobank-scale PRS construction and how external summary statistics can be integrated into such pipelines through its complementary method, uniLasso ES. Using the Python package Adelie, we implement polygenic risk score (PRS) algorithms for both continuous and binary outcomes.

We evaluate our methods on SNP data to demonstrate their gains in predictive performance and sparsity over existing approaches. Our experiments indicate that uniLasso achieves about 40% more sparsity than Lasso while maintaining comparable predictive performance. Furthermore, integrating external data into the individual-level model yields a noteworthy improvement in prediction accuracy. Among the individual-level sparse-regression models considered, uniLasso with external scores (uniLasso ES) achieves the highest point estimates for height, asthma, and CHD, and performs comparably to Elastic Net on BMI. Additionally, uniLasso ES retains substantially fewer non-zero coefficients than Lasso and far fewer than lassosum2, while PRS-CS maintains non-zero weights across the entire scored variant set. In the expanded comparison with summary-statistics-based methods, the FinnGen-UK Biobank-Meta PRS-CS configuration achieves the highest point estimates for BMI and CHD, but only by a narrow margin. However, when restricted to UK Biobank data alone, the strongest predictive performance was achieved by the individual-level sparse-regression models, rather than UK Biobank-only PRS-CS or lassosum2.

Across all settings, uniLasso’s enforced sparsity enables efficient computation in ultra-high-dimensional regimes while preserving, and often improving upon, the predictive accuracy of dense alternatives. It is particularly striking that for height, a canonical highly polygenic trait, our best uniLasso models rely on only about 34,000 non-zero SNPs out of more than one million candidates, yet still achieve test $R^{2}\approx 0.72$. Despite the extensive LD structure across the genome, uniLasso appears able to concentrate predictive signal onto a comparatively small subset of variants or LD blocks. These results suggest that, for prediction, much of the predictive signal can be captured by a substantially smaller active set than is used by dense methods, even for traits that are often described as highly polygenic.

From a predictive perspective, our results demonstrate that models with substantially smaller active sets can achieve predictive performance comparable to much denser approaches. Dense approaches such as PRS-CS remain valuable, especially when only GWAS summary statistics are available, while methods such as lassosum2 can induce some sparsity but typically retain substantially larger active sets than uniLasso ES. Our findings suggest that there is little predictive benefit in keeping millions of non-zero coefficients once LD and sample size are properly accounted for. In our experiments, models with only a few tens of thousands of non-zero variants achieved predictive performance comparable to substantially denser approaches.

Methods such as PRS-CS or infinitesimal-prior approaches effectively assume that every variant has a non-zero effect, but that most effects are very small. In the presence of LD, these dense models spread each signal over many correlated SNPs, so that each individual predictor carries a heavily shrunk, “flattened” effect size. This is attractive for aggregate prediction and some forms of inference, but it makes it difficult to identify which specific variants or LD blocks might be driving the score. Moreover, for individual-level risk prediction, reliance on many correlated tag variants may make the score sensitive to differences in LD structure across cohorts. In contrast, a sparse multivariate method concentrates weight on a subset of variants, yielding a less complex predictive model. While the selected variants should not be interpreted as causal, the reduced model complexity may facilitate downstream analyses by narrowing the set of variants considered for follow-up investigations, such as gene mapping and enrichment studies. Sparse representations can also provide computational advantages in downstream genomic analyses. For example, the recently proposed UltraLasso framework leverages a compact active set to enable efficient leave-one-chromosome-out (LOCO) prediction within a GPU-accelerated GWAS pipeline [20].

More broadly, our comparisons highlight a generic tension in LD-structured high-dimensional regression. Traditional GWAS is purely univariate, so each SNP’s marginal effect conflates its own contribution with that of its correlated neighbors. Dense multivariate models that do not explicitly promote sparsity tend to share this signal across entire correlated sets, yielding many predictors with tiny, flattened effect sizes. In contrast, the $\ell_{1}$ penalty and sign constraints in uniLasso force the model to concentrate the signal on a subset of SNPs within each LD block, reducing model complexity while maintaining out-of-sample predictive performance. We note, however, that although uniLasso does not require explicit LD-matrix modeling, it still relies on marginal effect estimates that reflect the underlying LD structure. Correlations among variants are implicitly accounted for through the individual-level regression model.

In genomic applications, linkage disequilibrium can induce substantial collinearity among SNPs, and the marginal, univariate regression coefficient for a SNP need not have the same sign as its conditional, multivariate coefficient. In particular, if Y=Xβ+ϵ, then the population univariate coefficient for feature *j* is proportional to $\gamma_{j}=\mathrm{Cov}(X_{j},Y)=\sum_{k}\Sigma_{jk}\beta_{k},$ where Σ=Cov(X). Thus, $\gamma_{j}$ reflects not only the direct effect $\beta_{j}$, but also the effects of SNPs in LD with *j*. Consequently, it is possible for $\gamma_{j}$ and $\beta_{j}$ to have opposite signs. This is an important limitation of any procedure that uses marginal effects as guidance. UniLasso is therefore not intended as a fine-mapping procedure for recovering causal SNP effects within a highly correlated LD block. Rather, its goal is prediction and stable variable selection, using the univariate associations as a form of directional regularization. In settings where the marginal and conditional signs are aligned, this can reduce variance and improve stability. However, when LD causes a marginal sign reversal, the uniLasso constraint or penalty can discourage the true conditional sign and may select another correlated SNP in the LD block instead.

There are a number of associated challenges that we plan to pursue as a natural consequence of this work. The existing uniLasso framework handles *K* = 2-class targets but not *K* > 2. This is an inherently difficult problem since the *K* coefficients for each feature are shift-invariant and hence their signs are not meaningful. We intend to develop and test new forms of uniLasso in the multiclass (multinomial) response context. A second extension concerns applying uniLasso to sets of SNPs found from linkage disequilibrium (LD)-based pre-clustering (e.g., via clumping or pruning) and studying the resulting false discovery rate [21]. There is recent work along these lines using Lasso which suggests this is a promising domain. Moreover, leave-one-chromosome-out (LOCO) cross-validation may help avoid signal leakage through LD and polygenic signals. Implementing LOCO folds within uniLasso could provide more reliable model assessment in genetic applications.

A practical limitation of the current uniLasso implementation is the memory required to store the (n×p) matrix of leave-one-out fitted values. While this can become prohibitive at biobank scale, the issue is largely computational rather than methodological. Existing large-scale penalized regression frameworks, such as the R package snpnet [3], employ screening rules and staged fitting procedures to avoid loading all variables into memory simultaneously. Incorporating similar memory-efficient screening strategies into the uniLasso Python pipeline is a promising direction for future work and could substantially reduce memory requirements while preserving the underlying statistical procedure. In settings where external summary statistics are available, the uniLasso ES formulation already mitigates this issue by avoiding construction of the full LOO matrix.

Our evaluation uses held-out samples from the UK Biobank rather than fully independent external cohorts. Validation of uniLasso in external datasets remains an important direction for future work and would provide a more rigorous assessment of generalizability and robustness to cohort-specific differences. Such validation may, however, be constrained by access to individual-level data, as uniLasso relies on leave-one-out quantities derived from them.

Finally, we are interested in developing a version of uniLasso that simultaneously incorporates both LOO fitted values and external univariate scores. As of now, uniLasso and uniLasso ES each leverage only one of these information sources; a unified approach that blends them could yield further gains in stability, sparsity, and predictive accuracy. Such a method would be especially valuable in large-scale genomic settings like the UK Biobank.

## Supporting information

S1 Appendix Algorithmic Runtimes. (PDF)

S2 Appendix Memory Usage. (PDF)

S3 Appendix Comparison of uniLasso with PRS-CS and lassosum2. This supplementary note describes the comparison pipeline for PRS-CS and lassosum2 in the four prediction tasks. The note reports the cohort splits, variant set, external summary-statistic inputs, model-fitting procedures, and held-out test-set prediction results.
(PDF)

S1 Table Approximate computation times for model training across methods for continuous and binary phenotypes. Standard uniLasso includes an additional preprocessing stage in which the LOO fitted values and univariate coefficients are computed for each feature. PRS-CS and lassosum2 also require GWAS as input, which we consider as preprocessing.
(PDF)

S2 Table PR-AUC and calibration metrics for binary traits. Performance of uniLasso, Lasso, and uniLasso ES on asthma and CHD. Metrics are computed on the test set.
(PDF)

S3 Table Predictive Performance of Covariates. Reported values correspond to test *R*^2^ (continuous traits) and AUC (binary traits) for models including only covariates (age, sex, and the top 10 principal components). This provides the baseline predictive performance against which the incremental contribution of PRS is assessed. All methods performed similarly up to 3 decimal places.
(PDF)

S4 Table Summary of UK Biobank cohort split used for model fitting, validation, and held-out prediction evaluation. UKBB denotes the UK Biobank.
(PDF)

S5 Table Held-out test-set prediction performances for all methods. Continuous traits are evaluated using full-model *R*^2^; binary traits are evaluated using full-model AUC. Bold indicates the highest point estimate within each phenotype among the listed models.
(PDF)

S6 TableSelected PRS-CS configurations and held-out test-set confidence intervals.Selection of ϕ used validation-set performance.(PDF)

S7 Table Number of active variants with nonzero lassosum2 weights for each summary-statistic source. (PDF)

S8 Table lassosum2 held-out test-set prediction performance with 95% bootstrap confidence intervals. Continuous traits are evaluated using full-model *R*^2^; binary traits are evaluated using full-model AUC.
(PDF)

S9 Table Matched-sparsity clumping-and-thresholding (C + T) sensitivity analysis. C + T scores were constructed using UK Biobank-only and FinnGen-UK Biobank-Meta summary statistics, with the number of variants matched to the corresponding uniLasso active-set size. Full rows include age, sex, ten genetic principal components, and the C + T score; PRS-only rows use the C + T score alone.
(PDF)

S10 Table Incremental test-set predictive performance beyond covariates for each PRS method and phenotype. For height and BMI, the metric is *R*^2^; for asthma and CHD, the metric is AUC. UniLasso with external scores is denoted as uniLasso ES, and clumping-and-thresholding as C + T. PRS-CS is a Bayesian polygenic prediction method, while lassosum2 is a summary-statistics-based regression approach.
(PDF)

S1 Fig UniLasso stability across random data splits. Stability is defined as the proportion of shared selected features between pairs of models, computed over 45 pairwise comparisons across 10 independent 75–25 train-test splits. The boxplot summarizes the distribution of these stability values, with median and interquartile range shown. The average stability is 0.3579.
(TIF)
