# Supplementary material for: Univariate-guided sparse regression for Biobank-scale high-dimensional omics data
Source: PLoS Genet. 2026 Sep 23;22(9):e1012314. doi: 10.1371/journal.pgen.1012314 (PMC13623159; doi:10.1371/journal.pgen.1012314)
Supplement: Supplementary file 1 [file pgen.1012314.s001.pdf]

S1 Appendix. Algorithmic Runtimes. An important practical consideration is the training time required for each algorithm, summarized in S1 Table. All computations were performed on Stanford University's high-performance computing cluster, Sherlock. For continuous outcomes, the preprocessing stage of uniLasso, which computes the leave-one-out (LOO) fitted values and the univariate coefficients, takes roughly 90 minutes on 64 CPUs for a dataset of size roughly about 250,000 individuals and 710,000 SNPs. For binary responses, the preprocessing runtime increases to around 4 hours due to the additional iterative approximation required in the IRLS optimization.

Fitting the uniLasso model with Gaussian loss after the computation of the pretraining values required approximately 3.5 hours. This is slightly longer than the average of 2.5 hours for Lasso, which in turn is greater than the 1.5 hour average for uniLasso with external scores (uniLasso ES). The reduced runtime of uniLasso ES arises from its formulation, which preserves the sparsity of the SNP matrix rather than populating it with dense LOO fitted values, as in the standard uniLasso implementation.

Training becomes even quicker when modeling binary outcomes. Under the same parameter configurations, Lasso finishes fitting in approximately 85 minutes, while uniLasso ES converges in only about 35 minutes. This exceptionally fast runtime further highlights the advantages of the uniLasso ES formulation. On the other hand, standard uniLasso requires an additional 5.5 hours of training after computing the necessary univariate and LOO estimates. With appropriate computational resources, for example GPU acceleration, this time could be substantially reduced. This is a direction we are actively pursuing. Nonetheless, the longer runtime is offset by the interpretability and unprecedented sparsity of the resulting model.

Across all response types, uniLasso with external scores achieves the fastest average training time among sparsity inducing competitors. This highlights the advantage of leveraging external information within the uniLasso framework, which reduces the need for intensive pretraining computations.

The PRS-CS pipeline involves substantial preprocessing and data formatting before the main model can be run, and additional steps are required afterward to aggregate the scores into a usable test metric. On Stanford's Sherlock cluster, the primary PRS-CS Python command required roughly 5.5 hours for continuous phenotypes and 2.5 hours for binary phenotypes (S1 Table). PRS-CS requires GWAS summary statistics as input, however, so we first run a genome-wide association study to generate the necessary single-variant effect estimates. This preprocessing stage showed the opposite pattern: about 1 hour for continuous responses versus 5 hours for binary. Overall, the end-to-end runtimes were similar, totaling approximately 6.5 hours and 7.5 hours for continuous and binary phenotypes respectively.

lassosum2 requires the same GWAS preprocessing pipeline as PRS-CS. Following preprocessing, however, runtimes were substantially shorter than those of the other methods considered. At the matched 750,510-variant scale, the total runtime per phenotype was approximately 20 minutes, including summary-statistic preprocessing, lassosum2 model fitting, and PLINK2 scoring. While this computational efficiency is a notable advantage, lassosum2 generally exhibited lower predictive performance than the competing methods and did not offer a substantial sparsity advantage.
