# Supplementary material for: Univariate-guided sparse regression for Biobank-scale high-dimensional omics data
Source: PLoS Genet. 2026 Sep 23;22(9):e1012314. doi: 10.1371/journal.pgen.1012314 (PMC13623159; doi:10.1371/journal.pgen.1012314)
Supplement: Supplementary file 2 [file pgen.1012314.s002.pdf]

S2 Appendix. Memory Usage.

Our compute instances use a cluster of Intel(R) Xeon(R) E5-2640 v4 CPUs with 64 total cores and ∼3TB RAM. Methods were run using 64 threads.

We applied standard preprocessing across all methods, including SNP quality control (e.g., minor allele frequency filtering) and population restriction (see Results section for more details). All methods used the same underlying feature matrix for each phenotype. UniLasso requires additional preprocessing to compute univariate fits and their corresponding leave-one-out (LOO) fitted values. In contrast, uniLasso-ES avoids these computations but requires alignment of external summary statistics, including matching variants and ensuring consistent allele coding and effect directions.

Genotype data were quality-controlled and encoded as a sparse matrix $X \in \mathbb{R}^{n \times p}$. Covariates (age, sex, and 10 principal components) were concatenated to the feature matrix. Because of the amount of SNPs being analyzed, our feature matrix has $n \approx 336,000$ and $p \approx 1.06$ million. All Lasso-based methods (Lasso, Elastic Net, and uniLasso variants) were fit using the same underlying matrix X, ensuring that baseline memory usage was identical across methods.

For Lasso and Elastic Net, no additional large-scale data structures were required beyond the feature matrix. As a result, peak memory usage was dominated by storage of X, which required approximately 82 GB.

In contrast, uniLasso requires the construction of a dense matrix of LOO fitted values. This matrix has the same dimensions as X but due to its dense nature, incurs a substantially larger memory cost. Each uniLasso run required approximately 1.3 TB of additional memory, depending on the dataset size (training vs. training+validation). When both runs were executed within a single session, peak memory usage reached approximately 2.7 TB. Since memory usage is dominated by storage of the feature matrix and LOO fitted values, it is therefore largely independent of whether the phenotype is continuous or binary.

While this represents a substantial increase relative to standard Lasso, it reflects the cost of explicitly leveraging out-of-sample (LOO) predictive structure. Notably, these computations remain feasible on modern high-memory compute nodes, and the method completes within a number hours in our implementation (see S1 Appendix).

On the other hand, an advantage of uniLasso with external scores (uniLasso ES) is that it avoids explicit construction of the LOO matrix. By incorporating external univariate coefficients directly into the optimization objective, the method eliminates the need for storing LOO features. As a result, the full pipeline has a peak memory usage of approximately 96 GB, including storage of X as well as generation, harmonization, and integration of external summary statistics. This represents only a modest increase over the base matrix storage (82 GB), while retaining the predictive benefits of the uniLasso framework.
