# Supplementary material for: Univariate-guided sparse regression for Biobank-scale high-dimensional omics data
Source: PLoS Genet. 2026 Sep 23;22(9):e1012314. doi: 10.1371/journal.pgen.1012314 (PMC13623159; doi:10.1371/journal.pgen.1012314)
Supplement: Supplementary file 3 [file pgen.1012314.s003.pdf]

S3 Appendix. Comparison of uniLasso with PRS-CS and lassosum2.

The four phenotypes were standing height and BMI (for continuous-trait prediction), and asthma and CHD (for binary-trait prediction). All prediction models included age, sex, and the first ten genetic principal components as covariates. The UK Biobank analysis sample was split into training, validation, training-plus-validation, and held-out test sets (see S4 Table). The held-out test set was used only for final prediction evaluation.

For the external-summary-statistic analyses, the corresponding FinnGen Release 12 phenotypes were matched to the same four endpoints; endpoint definitions are available through the FinnGen clinical endpoint repository (link). The PRS-CS and lassosum2 analyses used a matched set of 750,510 HapMap3 variants available in UK Biobank after common-variant quality control. Of these, 145,510 variants were directly available from the genotyping array and 605,000 were available from imputation; variants were required to pass MAF ≥ 0.01 and INFO > 0.8 where applicable. Of these, the FinnGen Release 12 summary statistics contained 735,142 variants for height and BMI and 733,574 variants for asthma and CHD, corresponding to an approximate retention rate of 98.5%.

PRS-CS was evaluated in three configurations. In the FinnGen-only configuration, PRS-CS was run using FinnGen Release 12 summary statistics, after which the downstream covariate-plus-PRS model was fit in the UK Biobank training-plus-validation cohort and evaluated in the test cohort. In the UK Biobank-only configuration, GWAS was run in the 249,711-sample UK Biobank training cohort; the downstream covariate-plus-PRS model was fit on the 20,000-sample validation cohort and evaluated on the test cohort. In the FinnGen-UK Biobank-Meta configuration, FinnGen Release 12 summary statistics were meta-analyzed with UK Biobank training-only GWAS summary statistics using inverse-variance weighting; the downstream covariate-plus-PRS model was then fit in the validation cohort and evaluated in the test cohort. For continuous traits, FinnGen effect estimates and standard errors were rescaled to the UK Biobank phenotype scale before meta-analysis.

For each PRS-CS configuration, the global shrinkage parameter ϕ was selected using validation-set performance from $\{\text{auto}, 10^{-2}, 10^{-4}, 10^{-6}\}$. PRS-CS used the standard European HapMap3 LD reference panel. PRS values were computed in UK Biobank using PLINK2 `--score`. Confidence intervals for PRS-CS rows were computed as nonparametric percentile intervals from 1,000 bootstrap resamples of the held-out test set.

lassosum2 was included as an additional summary-statistic PRS comparator using the same matched 750,510-variant set. The UKBB-only version used UK Biobank GWAS summary statistics, and the external-information version used FinnGen-UK Biobank meta-analyzed summary statistics. The lassosum2 grid included $\delta \in \{0.001, 0.01, 0.1, 1\}$ and a 30-point λ grid, with tuning parameters selected using validation-set performance.

As an additional sparse summary-statistic baseline, we evaluated clumping-and-thresholding (C+T) using the UK Biobank-only and FinnGen-UK Biobank-Meta summary-statistic sources. The number of selected variants was matched to the corresponding uniLasso active-set size. Full-model and PRS only metrics are reported in S9 Table.

Across the frozen prediction-model configurations, the highest point estimates were observed for uniLasso-ES for height ($R^2 = 0.716$) and asthma (AUC $= 0.623$), and for FinnGen-UK Biobank-Meta PRS-CS for BMI ($R^2 = 0.129$) and CHD (AUC $= 0.766$). In the UKBB-only comparison, the uniLasso-ES point estimates were higher than the UKBB-train-only PRS-CS point estimates for all four phenotypes. Adding FinnGen Release 12 summary statistics to PRS-CS increased the BMI and CHD point estimates relative to the UKBB-train-only PRS-CS configuration. The full set of point estimates,

including lassosum2 comparator rows, is shown in S5 Table.

The selected PRS-CS shrinkage parameter differed by phenotype and GWAS source. For the FinnGen-UK Biobank-Meta configuration, the selected value was $\phi = 10^{-2}$ for height, BMI, and asthma, and $\phi =$ auto for CHD. Selected PRS-CS configurations and bootstrap confidence intervals are provided in S6 Table. The lassosum2 confidence intervals and active-variant counts are shown in S8 Table and S7 Table respectively.
