# Supplementary material for: Univariate-guided sparse regression for Biobank-scale high-dimensional omics data
Source: PLoS Genet. 2026 Sep 23;22(9):e1012314. doi: 10.1371/journal.pgen.1012314 (PMC13623159; doi:10.1371/journal.pgen.1012314)
Supplement: Supplementary file 4 [file pgen.1012314.s004.pdf]

**Method / Stage**	**Cont. Response**	**Bin. Response**
Preprocessing		
uniLasso (pretraining)	90 min	4 hr
PRS-CS (GWAS preprocessing)	1 hr	5 hr
lassosum2 (GWAS preprocessing)	1 hr	5 hr
Training Time		
Lasso	2.5 hr	85 min
uniLasso (after pretraining)	3 hr	5 hr
uniLasso ES	1.5 hr	35 min
PRS-CS	5.5 hr	2.5 hr
lassosum2	20 min	20 min
