# Supplementary material for: Univariate-guided sparse regression for Biobank-scale high-dimensional omics data
Source: PLoS Genet. 2026 Sep 23;22(9):e1012314. doi: 10.1371/journal.pgen.1012314 (PMC13623159; doi:10.1371/journal.pgen.1012314)
Supplement: Supplementary file 5 [file pgen.1012314.s005.pdf]

	PR-AUC			Calibration		
	**uniLasso**	**Lasso**	**uniLasso ES**	**uniLasso**	**Lasso**	**uniLasso ES**
**Asthma**	0.2021	0.2029	0.1993	0.1123	0.1123	0.1123
**CHD**	0.1206	0.1186	0.1201	0.0383	0.0383	0.0383
