# Supplementary material for: Univariate-guided sparse regression for Biobank-scale high-dimensional omics data
Source: PLoS Genet. 2026 Sep 23;22(9):e1012314. doi: 10.1371/journal.pgen.1012314 (PMC13623159; doi:10.1371/journal.pgen.1012314)
Supplement: Supplementary file 6 [file pgen.1012314.s006.pdf]

**Phenotype**	**Lasso**$_{\text{cov}}$	**uniLasso**$_{\text{cov}}$	**uniLasso ES**$_{\text{cov}}$
Height	0.536	0.536	0.536
BMI	0.010	0.010	0.010
Asthma	0.530	0.530	0.530
CHD	0.745	0.745	0.745
