# Supplementary material for: Univariate-guided sparse regression for Biobank-scale high-dimensional omics data
Source: PLoS Genet. 2026 Sep 23;22(9):e1012314. doi: 10.1371/journal.pgen.1012314 (PMC13623159; doi:10.1371/journal.pgen.1012314)
Supplement: Supplementary file 7 [file pgen.1012314.s007.pdf]

Block	Sample Size	Use
**Training**	249,711	Individual-level model fitting; UKBB training-only GWAS for PRS-CS.
**Validation**	20,000	Tuning-parameter selection; PRS calibration for UKBB training-only GWAS.
**Training + validation**	269,711	Final individual-level sparse-regression model fitting; PRS calibration when the GWAS source was external.
**Held-out test**	67,427	Prediction evaluation.
