# Supplementary material for: Univariate-guided sparse regression for Biobank-scale high-dimensional omics data
Source: PLoS Genet. 2026 Sep 23;22(9):e1012314. doi: 10.1371/journal.pgen.1012314 (PMC13623159; doi:10.1371/journal.pgen.1012314)
Supplement: Supplementary file 8 [file pgen.1012314.s008.pdf]

Model	Height R^2	BMI R^2	Asthma AUC	CHD AUC
Covariates only	0.536	0.010	0.530	0.745
Lasso	0.713	0.119	0.619	0.758
Elastic Net	0.712	0.122	0.620	0.758
uniLasso	0.707	0.103	0.620	0.757
uniLasso-ES	**0.716**	0.118	**0.623**	0.760
PRS-CS, FinnGen-only	0.643	0.087	0.599	0.763
PRS-CS, UK Biobank-train-only	0.675	0.112	0.609	0.757
PRS-CS, FinnGen-UK Biobank-Meta	0.681	**0.129**	0.617	**0.766**
lassosum2, UK Biobank	0.676	0.105	0.586	0.751
lassosum2, FinnGen-only	0.645	0.086	0.603	0.762
lassosum2, FinnGen-UK Biobank-Meta	0.675	0.122	0.591	0.757
