# Supplementary material for: Univariate-guided sparse regression for Biobank-scale high-dimensional omics data
Source: PLoS Genet. 2026 Sep 23;22(9):e1012314. doi: 10.1371/journal.pgen.1012314 (PMC13623159; doi:10.1371/journal.pgen.1012314)
Supplement: Supplementary file 9 [file pgen.1012314.s009.pdf]

PRS-CS configuration	Phenotype	Selected ϕ	Test metric	95% CI lower	95% CI upper
FinnGen-only	Height	auto	0.643	0.639	0.647
FinnGen-only	BMI	10^{-2}	0.087	0.083	0.091
FinnGen-only	Asthma	auto	0.599	0.593	0.605
FinnGen-only	CHD	10^{-4}	0.763	0.754	0.772
UK Biobank-train-only	Height	10^{-2}	0.675	0.671	0.679
UK Biobank-train-only	BMI	10^{-2}	0.112	0.107	0.117
UK Biobank-train-only	Asthma	auto	0.609	0.603	0.615
UK Biobank-train-only	CHD	10^{-4}	0.757	0.749	0.767
FinnGen-UK Biobank-Meta	Height	10^{-2}	0.681	0.677	0.685
FinnGen-UK Biobank-Meta	BMI	10^{-2}	0.129	0.124	0.133
FinnGen-UK Biobank-Meta	Asthma	10^{-2}	0.617	0.610	0.623
FinnGen-UK Biobank-Meta	CHD	auto	0.766	0.758	0.776
