# Supplementary material for: Univariate-guided sparse regression for Biobank-scale high-dimensional omics data
Source: PLoS Genet. 2026 Sep 23;22(9):e1012314. doi: 10.1371/journal.pgen.1012314 (PMC13623159; doi:10.1371/journal.pgen.1012314)
Supplement: Supplementary file 10 [file pgen.1012314.s010.pdf]

lassosum2 Summary-Statistic Source	Height	BMI	Asthma	CHD
UK Biobank-only	100,104	99,637	99,390	102,163
FinnGen-only	87,428	127,816	31,741	22,720
FinnGen-UK Biobank-Meta	140,423	127,847	139,766	274,158
