# Supplementary material for: Univariate-guided sparse regression for Biobank-scale high-dimensional omics data
Source: PLoS Genet. 2026 Sep 23;22(9):e1012314. doi: 10.1371/journal.pgen.1012314 (PMC13623159; doi:10.1371/journal.pgen.1012314)
Supplement: Supplementary file 11 [file pgen.1012314.s011.pdf]

lassosum2 Summary-Statistic Source	Phenotype	Test metric [95% CI]
UK Biobank-only	Height	0.676 [0.672, 0.679]
UK Biobank-only	BMI	0.105 [0.100, 0.109]
UK Biobank-only	Asthma	0.586 [0.579, 0.592]
UK Biobank-only	CHD	0.751 [0.743, 0.760]
FinnGen-only	Height	0.645 [0.641, 0.649]
FinnGen-only	BMI	0.086 [0.082, 0.090]
FinnGen-only	Asthma	0.603 [0.596, 0.609]
FinnGen-only	CHD	0.762 [0.753, 0.771]
FinnGen-UK Biobank-Meta	Height	0.675 [0.671, 0.679]
FinnGen-UK Biobank-Meta	BMI	0.122 [0.117, 0.126]
FinnGen-UK Biobank-Meta	Asthma	0.591 [0.585, 0.597]
FinnGen-UK Biobank-Meta	CHD	0.757 [0.748, 0.766]
