# Supplementary material for: Univariate-guided sparse regression for Biobank-scale high-dimensional omics data
Source: PLoS Genet. 2026 Sep 23;22(9):e1012314. doi: 10.1371/journal.pgen.1012314 (PMC13623159; doi:10.1371/journal.pgen.1012314)
Supplement: Supplementary file 12 [file pgen.1012314.s012.pdf]

C+T Source	Model	Height R^2	BMI R^2	Asthma AUC	CHD AUC
UKBB-only	Full	0.647	0.091	0.594	0.751
UKBB-only	PRS-only	0.110	0.082	0.590	0.561
FG-UKBB-Meta	Full	0.651	0.100	0.605	0.759
FG-UKBB-Meta	PRS-only	0.114	0.091	0.601	0.588
