# Supplementary material for: Univariate-guided sparse regression for Biobank-scale high-dimensional omics data
Source: PLoS Genet. 2026 Sep 23;22(9):e1012314. doi: 10.1371/journal.pgen.1012314 (PMC13623159; doi:10.1371/journal.pgen.1012314)
Supplement: Supplementary file 13 [file pgen.1012314.s013.pdf]

Phenotype	Lasso	uniLasso	uniLasso ES	PRS-CS	lassosum2	C+T
Height	0.177	0.171	0.180	0.145	0.139	0.115
BMI	0.109	0.093	0.108	0.120	0.113	0.090
Asthma	0.089	0.090	0.093	0.087	0.061	0.075
CHD	0.013	0.012	0.015	0.021	0.012	0.014
