# Supplementary material for: Univariate-guided sparse regression for Biobank-scale high-dimensional omics data
Source: PLoS Genet. 2026 Sep 23;22(9):e1012314. doi: 10.1371/journal.pgen.1012314 (PMC13623159; doi:10.1371/journal.pgen.1012314)
Supplement: Supplementary file 14 [file pgen.1012314.s016.pdf]

6th June, 2026

We thank the editor and Reviewers for very constructive comments and for the opportunity to revise our manuscript. We have now made every effort to incorporate the comments into the revised manuscript. We have addressed the priority items as follows:

Reviewers' comments:

Reviewer 1

Reviewer #1: This paper presents a method to construct polygenic risk score (PRS) based on uniLasso algorithm. UniLasso is a two-step method that first performs univariate feature screening and then applies Lasso regression on the selected variables to build a sparse multivariate model. Overall, I think the method is solid and reflects the latest developments by the Lasso authors in genomic prediction. Below are my comments.

1. Arguably, one of the central challenges in genomic prediction is handling collinearity among features induced by linkage disequilibrium (LD). While I appreciate the advancement of UniLasso in promoting sign consistency between univariate and multivariate coefficient estimates, I was wondering how uniLasso behaves to infer true genetic effects among SNPs in LD, as univariate regression coefficients can have opposite signs from the true multivariate effects (Y = X\beta + \epsilon, \hat{\beta_j} = (x_j^T x_j)^-1 x_j^T y, E[\hat{\beta_j}] = (x_j^T x_j)^-1 x_j^T X \beta which can have different sign with \beta_j). A small simulation example or discussion could help illustrate this point.

We thank the reviewer for raising this important point. We agree that in genomic applications, linkage disequilibrium can induce substantial collinearity among SNPs, and that the marginal, univariate regression coefficient for a SNP need not have the same sign as its conditional, multivariate coefficient. In particular, if (Y=X\beta+\epsilon), then the population univariate coefficient for feature (j) is proportional to
[
\gamma_j = \operatorname{Cov}(X_j,Y)
= \sum_k \Sigma_{jk}\beta_k,
]
where (\Sigma=\operatorname{Cov}(X)). Thus (\gamma_j) reflects not only the direct effect (\beta_j), but also the effects of SNPs in LD with (j). Consequently, it is possible for (\gamma_j) and (\beta_j) to have opposite signs.

This is an important limitation of any procedure that uses marginal effects as guidance. UniLasso is therefore not intended as a fine-mapping procedure for recovering causal SNP effects within a highly correlated LD block. Rather, its goal is prediction and stable variable selection, using the univariate associations as a form of directional regularization. In settings where the marginal and conditional signs are aligned, this can reduce variance and improve stability. However, when LD causes a marginal sign reversal, the UniLasso constraint or penalty can discourage the true conditional sign and may select another correlated SNP in the LD block instead.

We have added this important point to the manuscript

2. What is the performance of elastic net for Fig. 1?

This is a helpful suggestion. We have included Elastic Net as an additional baseline and report its results for each phenotype alongside the other methods in Fig. 1 and Table 1.

3. The cut-off of minor allele frequency to define common SNPs to build PRS model is typically MAF > 0.01 or MAF > 0.05.

Thank you very much for pointing this out, you are certainly right that this is most typical. In our setting, we intentionally chose a more permissive threshold to remain inclusive of low-frequency variants that may still carry predictive signals at biobank scale. That said, we agree that the choice of MAF threshold is important. We have clarified this design decision in the manuscript, please see lines 225-226.

4. The statistical foundation of PRS-CS is horseshore prior (https://academic.oup.com/biomet/article/97/2/465/219397?login=true). Although PRS-CS is Bayesian and not forcing the coefficients to be exactly zero, I think it does encourage sparsity in the coefficients by shrinking most of them towards 0 (Line 387-388). There are also some caveats when comparing with PRS-CS: 1) PRS-CS typically restricts analysis to HapMap3 SNPs, so a fair comparison requires using the same SNP set for both UniLasso and PRS-CS; 2) PRS-CS has a tuning parameter phi that can often be tuned to improve the performance.

We thank the reviewer for this helpful comment. We have revised the PRS-CS comparison to address both issues. First, we now describe PRS-CS more precisely as a Bayesian continuous-shrinkage method that shrinks effect sizes but does not induce exact sparsity in the same sense as Lasso-based methods. Second, we rebuilt the PRS-CS comparison using a matched HapMap3-compatible variant set. The PRS-CS and lassosum2 analyses used 750,510 HapMap3 variants available in UK Biobank after common-variant quality control. Of these, 145,510 variants were directly available from the genotyping array and 605,000 were available from imputation; variants were

required to pass MAF >= 0.01 and INFO > 0.8 where applicable. This corrected the variant-set discrepancy in the original comparison and avoids penalizing PRS-CS through poor overlap with its LD reference panel. We also tuned the PRS-CS global shrinkage parameter phi using validation-set performance over {auto, 1e-2, 1e-4, 1e-6}, and report the selected values and held-out test-set confidence intervals in the revised supplementary tables.

5. It may be helpful to discuss whether the authors think it is possible to fit UniLasso entirely based on external summary statistics since 1) the individual level data y and X are not accessible for many traits, 2) the sample size of external summary statistics is often much larger than biobank. Currently the most widely used lasso approach for polygenic prediction is lassosum (https://pubmed.ncbi.nlm.nih.gov/28480976/), which fits lasso based on summary statistics.

This is an important point. In its current form, uniLasso is an individual-level regression method and requires access to X and y to fit the multivariate model. In particular, the method relies on leave-one-out (LOO) fitted values, which depend on individual-level data and cannot be recovered from summary statistics alone. As a result, we do not believe that uniLasso can be directly reduced to a fully summary-statistics-based formulation. Extending uniLasso in this direction would likely require a substantially different formulation and is an interesting avenue for future work. We have included a sentence in the manuscript to address your helpful observation (see lines 585-587).

To address the reviewer's suggestion, we added lassosum2 as a summary-statistics-based comparator. We evaluated lassosum2 using the same 750,510 HapMap3-compatible variant set and the same summary-statistic sources used for PRS-CS, including UK Biobank-only, FinnGen-only, and FinnGen-UK Biobank-Meta configurations. The selected lassosum2 active variant counts and held-out test-set confidence intervals are now reported in the supplementary tables.

6. What is the memory usage of uniLasso on the real data compared with other approaches?

We thank the reviewer for this helpful question. In addition to wall-clock time, we now report peak memory usage for all methods. These results highlight that while uniLasso incurs substantial additional memory due to construction of the LOO matrix, uniLasso-ES avoids this cost and has memory usage comparable to standard Lasso-based approaches. Please see Appendix S2 for our discussion of memory usage.

7. I cannot find the link to the package.

The package is now publicly available on GitHub (link), although it is still under active development. For reproducibility, the folder titled “Examples with Functions Defined” contains the pipeline used prior to constructing the package, and the same functionality is now accessible through the PRS-Lasso package.

Reviewer #2: PGENETICS-D-26-00283

Univariate-guided sparse regression for Biobank-scale high-dimensional omics data

In this paper, Richland and colleagues adapted the Univariate-Guided Sparse Regression (uniLasso) method to ultra-high-dimensional settings, and demonstrated its application to polygenic prediction.
The authors showed that uniLasso achieves comparable predictive performance to standard Lasso while yielding a sparser solution, and further extended the framework by incorporating external GWAS summary statistics, which improves prediction accuracy.
Overall, the methodological development is sound. However, there is a notable gap between the evaluation framework used in this study and current best practices in statistical genetics, which I detail below.

- Comparison with PRS-CS
The comparison between uniLasso and PRS-CS lacks critical detail regarding variant selection.
The manuscript reports using 1,080,968 variants, but does not specify how these were selected.
This number is much smaller than a typical GWAS on imputed variants (often 8–10 million in UK Biobank) but seems to be larger than the set of directly genotyped variants (~800K in UK Biobank if I recall correctly).

Thank you for pointing this out. The 1,080,968 variants correspond to a quality-controlled subset of UK Biobank variants consisting primarily of directly genotyped SNPs, together with CNV calls and HLA alleles derived from the array data. This set was obtained after standard preprocessing and quality-control procedures, including filtering based on missingness, minor allele frequency, and Hardy–Weinberg equilibrium. As a result, the number of variants is larger than the directly genotyped SNP array alone, but substantially smaller than the full imputed variant panel typically used in large-scale GWAS analyses.

We have clarified this in the manuscript (Results Section: UK Biobank analysis).

This is critical technical detail because PRS-CS uses ~1.2M HapMap3 variants for PRS construction, most of which are available in well-imputed GWAS.
If the authors used a randomly selected or genotyped set of ~1M variants, many variants may not overlap with the PRS-CS reference panel.
This would significantly reduce the effective number of variants used for PRS-CS (possibly <200K), leading to an unfair comparison.
Please align with standard practice by either using full GWAS summary statistics or restricting analyses to HapMap3 variants.

In addition, the manuscript should provide detailed information on sample and variant QC, case/control definitions, and the number of variants used for PRS construction for each method.

We thank the reviewer for identifying this issue. We have clarified that the 1,080,968 variants used for the individual-level Lasso, Elastic Net, uniLasso, and uniLasso-ES analyses are a quality-controlled UK Biobank array-derived variant set, consisting primarily of directly genotyped SNPs together with array-derived CNV and HLA features. We retained this array-based individual-level feature set to maintain comparability with previous large-scale sparse-regression work in UK Biobank. For PRS-CS and lassosum2, however, we revised the comparison to follow standard summary-statistic PRS practice and to match the HapMap3-compatible variant universe used by these methods. Specifically, we used 750,510 HapMap3 variants available in UK Biobank after common-variant quality control, including 145,510 directly array-available variants and 605,000 imputed variants. The reduction from the approximately 1.08 million individual-level variants to the PRS analysis set is therefore explained by the HapMap3 restriction and common-variant quality control. We also added detailed descriptions of the UK Biobank cohort split, phenotype definitions, variant counts, FinnGen overlap, and PRS calibration strategy in the revised manuscript and supplementary tables.

- Sparsity and interpretation
The manuscript emphasizes sparsity as a key advantage of uniLasso. However:
(i) In polygenic prediction, the primary objective is to maximize predictive accuracy rather than interpretability. People usually don't care about sparseness and don't interpret selected variants.
(ii) It is unclear whether reducing the number of nonzero coefficients by uniLasso (e.g., from 30K to 20K variants) meaningfully improves interpretability in practice.
In vast majority of applications, thousands or tens of thousands variants will still be selected.
(iii) The authors didn't interpret selected variants and provided any concrete demonstrations of interpretability gains.

This is a helpful observation, and we agree that predictive accuracy is typically the primary objective in polygenic prediction. Our goal is not to replace dense approaches in such settings, but to provide an alternative that achieves comparable accuracy while yielding substantially sparser models.

To date, most widely used polygenic prediction methods produce dense solutions. An important contribution of this work is to demonstrate that substantial sparsity can be achieved without sacrificing predictive performance. This suggests that sparsity may be

a viable modeling objective, opening the possibility that it can be useful beyond purely predictive considerations.

We also agree that sparsity at the scale of tens of thousands of variants does not directly enable variant-level biological interpretation. Rather, we view sparsity as improving model simplicity, stability, and potential downstream usability (e.g., reduced storage, easier deployment, and more interpretable aggregation at the level of genomic regions or LD blocks), rather than identifying individual causal variants. That being said, while the selected variants should not be interpreted as causal, the reduced model complexity can facilitate downstream analyses by narrowing the set of variants considered in follow-up investigations, such as gene mapping and enrichment studies. We have clarified this point in the manuscript (see lines 530-532).

(iv and v) How does the feature selection in uniLasso compare with simply ranking and selecting lead variants from independent genomic loci, or thresholding Bayesian posterior effect sizes?

The authors should also compare uniLasso with clumping and thresholding, which selects lead variants from each genomic locus and can be as sparse and as 'interpretable' as one would like by varying the p-value threshold.

We thank the reviewer for this useful suggestion. We have added a matched-sparsity clumping-and-thresholding (C+T) sensitivity analysis. In this analysis, the number of selected variants was matched to the corresponding uniLasso active-set size, and C+T scores were constructed using both UK Biobank-only and FinnGen-UK Biobank-Meta summary-statistic sources. This simple pruning strategy yielded lower point estimates than uniLasso in seven of eight C+T Union or C+T Meta cells; relative to standard uniLasso, the differences ranged from -0.060 to +0.002 in R2/AUC units. Relative to uniLasso-ES, the C+T point estimates were lower in all eight cells. We report the full-model and PRS-only C+T results in a new supplementary table (S9 Table). For Bayesian posterior thresholding, we note that PRS-CS is already included as the Bayesian continuous-shrinkage comparator. We did not add a separate post hoc thresholding of PRS-CS posterior weights as a main analysis, because that would introduce an additional arbitrary sparsity-tuning step beyond the standard PRS-CS model.

(vi) One advantage of Bayesian shrinkage methods such as PRS-CS is that the SNP weights are more robust when applied to external datasets.
For example, a large number of PRS weights have been released via PGS Catalog, which allows for replication, application, and reuse of trained PRS model.
If weights concentrate on a small set of variants, and some of the variants are missing from target datasets, the predictive performance may decrease dramatically.

It would be useful to discuss the trade-offs between sparsity and robustness, given that highly sparse models may be sensitive to missing variants in external datasets.

We thank the reviewer for raising this important practical issue. We agree that dense Bayesian shrinkage methods can be attractive for broad reuse across heterogeneous target datasets, particularly when the goal is to release a transferrable PRS through resources such as the PGS Catalog. Our study has a different aim: it introduces and benchmarks a sparse individual-level modeling framework rather than releasing final clinical PRS weights. We have revised the Discussion to acknowledge the trade-off between sparsity and transferability. A highly sparse score may be more sensitive to missing variants in an external cohort, whereas dense scores may be more robust to incomplete variant coverage. For future development of transferrable sparse PRS, one could deliberately restrict the candidate variant set to common, well-covered variants available across target datasets before model fitting. Developing and externally validating such catalog-ready sparse PRS is important future work but is beyond the scope of the present methods paper.

(vii) From table 3, it looks that there is limited overlap of selected variants across Lasso-based methods, which raises concerns about feature stability and interpretability.

We agree that feature stability is an important consideration. To directly address this, we performed an explicit stability analysis based on repeated data splits. Specifically, we generated 10 independent 75/25 train/validation splits, fit uniLasso on each training set, selected the tuning parameter via validation R-squared, and then computed pairwise overlap across all model pairs. Please see the newly included Fig. S1, which displays the results of this stability analysis for uniLasso.

We note that in high-dimensional genetic data, limited exact overlap at the variant level is expected due to strong correlations (LD), where multiple nearby variants can act as interchangeable predictors. As a result, different splits may select different representatives from the same signal region without specifically affecting predictive performance.

- Individual-level data vs. summary statistics

The manuscript frames the requirement for individual-level data as a strength, but this may be viewed as a limitation given the limited accessibility of individual-level data for most large-scale GWAS. A more balanced discussion is warranted.
When incorporating external summary statistics (e.g., FinnGen), the authors should also benchmark against standard approaches that meta-analyze summary statistics from FinnGen and UKB GWAS and run PRS algorithms on the meta-GWAS summary statistics.

We thank the reviewer for this important suggestion. We have revised the manuscript to present the individual-level requirement in a more balanced way: it is central to the uniLasso modeling strategy, but it also means that the method cannot be applied when only summary statistics are available. To make the external-information comparison more complete, we added PRS-CS and lassosum2 analyses using FinnGen-only summary statistics, UK Biobank training-only GWAS summary statistics, and FinnGen-UK Biobank meta-analyzed summary statistics. The FinnGen-UK Biobank-Meta configuration is intentionally a strong comparator for standard PRS pipelines: it combines external FinnGen summary statistics with a UK Biobank training-only GWAS using inverse-variance weighting, constructs a PRS, calibrates the downstream covariate-plus-PRS model in validation samples, and evaluates the frozen model in the held-out test set. This benchmark is therefore more information-dense than a FinnGen-only PRS and directly addresses the reviewer's request for meta-analyzed summary-statistic comparisons.

- External validation

All evaluations are conducted within UK Biobank by splitting samples into training, validation, and testing sets.
Assessing performance in an independent external dataset would provide a more rigorous evaluation of generalizability and reduce concerns about overfitting.

This is a very good point and we agree that evaluation on an independent external dataset would strengthen the results. Unfortunately, access to such data is limited due to privacy constraints around individual-level genomic data.

To mitigate overfitting within this constraint, we adopted a strict train/validation/test split and ensured that all model selection (including tuning λ) is performed without access to the held-out test set. In addition, our uniLasso ES results incorporate external summary statistics (FinnGen), providing partial validation across datasets. This demonstrates that the method can effectively leverage information from independent sources, even when individual-level external data are unavailable.

We have revised the manuscript to avoid presenting the held-out UK Biobank test set as a fully external validation cohort. The goal of this paper is to introduce and benchmark the uniLasso framework and uniLasso-ES method, not to release final clinical PRS models. External validation of final frozen PRS models is an important next step, but it requires access to external individual-level genotype and phenotype data harmonized to the same endpoints. This is beyond the scope of the present methods paper and is now stated explicitly as a limitation. Please see lines 583-588.

- Causal variants

The authors claimed that sparse multivariate methods "concentrate weight on (or near) truly causal variants", but this is not justified by the analyses. I feel this is overstated. In statistical genetics, there exists a set of dedicated fine-mapping methods and evaluation metrics for causal inference.
The limited overlap of selected features between different types of Lasso methods raised additional concerns about whether selected variants are truly causal.
I think the authors should tune down on this point.

We thank the reviewer for this point and agree that our original phrasing was too strong. Our analyses do not establish that selected variants are causal, and we do not intend to make a fine-mapping claim.

We have revised the text to remove causal language and instead emphasize that sparse multivariate methods concentrate signal on a subset of variants, which may improve interpretability and robustness of individual risk estimates. Please see the amendment on lines 528-529.

- Evaluation metrics

The authors reported R2 and AUC of the combined model (covariates + PRS) rather than reporting the incremental R2 and AUC of PRS beyond covariates, which obscures the predictive value of PRS.

We thank the reviewer for this important clarification. We agree that reporting only the combined model can obscure the specific contribution of PRS. In response, we now explicitly report the performance of the covariate-only model (age, sex, and principal components). This isolates the predictive value attributable to genetic information. These results are now included in Table S3.

Please also report appropriate uncertainty estimates (e.g., standard errors or confidence intervals) for all predictions.

This is a helpful suggestion. We have added uncertainty estimates for the prediction metrics. For the individual-level sparse-regression models, standard errors are shown in Fig 1. For PRS-CS and lassosum2, we report 95% confidence intervals from 1,000 nonparametric bootstrap resamples of the held-out test set in the supplementary tables (S6 and S8 Tables respectively).

- Manuscript writing

The manuscript was written with a strong statistical flavor and in some places not for a genetic audience.

Please consider our revisions to improve clarity and accessibility for a broader genetics audience.

The use of examples from finance in the introduction is not aligned with the scope of PLoS Genetics.

Thank you for pointing this out to us, we have removed this example from the introduction.

The authors may consider either sending the manuscript to a statistical journal or tailoring the framing to better suit the target audience.

We have worked to frame the manuscript to better suit a genetics and statistical genetics audience.

Reviewer #3:

This manuscript proposes uniLasso and its extension, uniLasso ES, for sparse polygenic prediction in biobank-scale settings. The idea is interesting, and the method appears potentially useful, particularly because it avoids explicit LD-matrix construction and seems scalable to large individual-level datasets. The inclusion of both quantitative and binary traits is also a strength. That said, I think the manuscript would benefit from clearer methodological positioning, fairer benchmarking, and a more careful discussion of limitations.

Major comments

1. The methodological positioning should be clarified more explicitly.
A central point that should be made much clearer is that this is not a purely summary-statistics-based PRS method. Both uniLasso and uniLasso ES ultimately fit a multivariable model using individual-level target data. In uniLasso ES, external summary statistics are used to guide penalties and/or sign constraints, rather than to replace individual-level fitting.

This distinction matters because comparisons to methods such as PRS-CS are informative, but not fully apples-to-apples. PRS-CS is a summary-statistics-based framework that relies on an LD reference, whereas uniLasso ES is an individual-level regression framework that can additionally leverage external summary information. I recommend that the authors make this distinction much clearer in the Introduction, Methods, and Discussion, so that readers do not misinterpret the method class or its intended use case.

We thank the reviewer for this helpful clarification and agree that the distinction was not sufficiently emphasized. We have revised the Abstract, Introduction, Methods, and Discussion to make clear that both uniLasso and uniLasso-ES are individual-level regression methods. In uniLasso-ES, external summary statistics guide the individual-level fit through penalty weights and sign constraints; they do not replace target-cohort model fitting. We also now distinguish this framework from summary-statistic PRS methods such as PRS-CS and lassosum2, which first build a raw PRS from GWAS summary statistics and then require downstream calibration before being used as phenotype-scale prediction models.

2. The distinction between uniLasso and uniLasso ES should be made much clearer.
Based on the reported results and runtime benchmarks, the strongest practical contribution appears to come from uniLasso ES rather than standard uniLasso. Standard uniLasso seems substantially more computationally expensive, especially for

binary traits. The manuscript should therefore distinguish more clearly between the methodological/statistical contribution of standard uniLasso and the practical/computational contribution of uniLasso ES.

We entirely agree that the distinction between uniLasso and uniLasso ES should be made clearer. We have revised the manuscript to more explicitly separate the methodological contribution of uniLasso from the practical advantages of uniLasso ES. In particular, we emphasize that uniLasso provides the core modeling framework, while uniLasso ES extends this framework by incorporating external summary statistics, which often improves predictive performance and can support more efficient modeling in high-dimensional settings.

3. Comparisons involving uniLasso ES do not appear fully information-matched. uniLasso ES leverages external GWAS summary statistics, whereas some reported baselines appear to rely primarily on target-cohort information. This makes it difficult to distinguish gains due to the modeling strategy itself from gains due to access to additional external information.
In my view, uniLasso ES should ideally be compared against methods given access to the same external data, such as PRS-CS or lassosum/lassosum2 run on the same external summary statistics, and, if feasible, methods based on meta-analyzed internal plus external summary statistics. Without such information-matched comparisons, the practical advantage of uniLasso ES is difficult to assess cleanly.

We thank the reviewer for this important suggestion. We have now added information-matched summary-statistic PRS comparisons using PRS-CS and lassosum2. Specifically, we evaluated both methods using FinnGen-only summary statistics, UK Biobank training-only GWAS summary statistics, and FinnGen-UK Biobank meta-analyzed summary statistics. The FinnGen-UK Biobank-Meta configuration is intentionally a strong comparator: it uses external FinnGen summary statistics, adds a UK Biobank training-only GWAS through inverse-variance meta-analysis, constructs the PRS from the combined summary statistics, calibrates the downstream covariate-plus-PRS model in validation samples, and evaluates the frozen model in the held-out test set. This comparison is therefore more information-dense than a FinnGen-only PRS and directly addresses the concern that uniLasso-ES had access to external summary statistics while the original comparator did not. At the same time, uniLasso-ES and meta-analyzed PRS methods use external information in different ways. The FinnGen-UK Biobank-Meta PRS-CS analysis directly increases summary-statistic power by combining GWAS evidence before PRS construction, whereas uniLasso-ES uses external effects to guide penalty weights and sign constraints within an individual-level sparse regression model. Thus, the comparison evaluates two different strategies for using external information rather than identical

uses of the same input.

4. The manuscript should be positioned more clearly relative to prior hybrid approaches. The paper would benefit from a more explicit discussion of prior work on combining individual-level data with external GWAS summary statistics, particularly Albiñana et al. (AJHG 2021, Leveraging both individual-level genetic data and GWAS summary statistics increases polygenic prediction), which compared meta-GWAS, SCT, and meta-PRS. At minimum, the authors should clarify how uniLasso ES differs conceptually and methodologically from these earlier hybrid/data-combining approaches.

We thank the reviewer for this helpful suggestion and agree that positioning relative to prior hybrid approaches should be clarified. UniLasso ES is unique in that it incorporates external summary statistics directly within a single individual-level regression model by using them to guide penalty weights and/or sign constraints. This allows external information to influence variable selection and coefficient estimation within a unified sparse model, rather than combining separate scores post hoc. Please see lines 113-116 for where we explicitly discuss this.

5. “No explicit LD matrix” is a practical advantage, but the LD-related discussion should be more careful. The fact that the method does not require explicit LD-matrix modeling is attractive. However, it still relies on marginal effect information, which is itself influenced by LD. The Discussion should therefore acknowledge more explicitly the possible sensitivity of the approach to LD mismatch, ancestry differences, phenotype-definition differences, and covariate-adjustment differences between external and target datasets.

We agree and have revised the Discussion accordingly. We now state that although uniLasso does not require explicit LD-matrix construction, it still relies on marginal association information that reflects the LD structure of the data. As a result, performance may be sensitive to LD mismatch, ancestry differences, phenotype definitions, and covariate adjustment differences between external and target datasets. We also clarified that uniLasso should not be interpreted as a fine-mapping method for identifying causal variants within LD blocks. Please see lines 540-542.

6. The manuscript lacks truly external out-of-sample validation, and generalizability therefore remains unclear.
The train/validation/test split within UK Biobank is reasonable for method development, but it remains an internal held-out evaluation within the same cohort. For PRS methods, an important practical question is whether the model transports well to independent external cohorts. This is especially relevant here because the paper emphasizes

practical prediction and the use of external summary information. However, the use of external summary statistics is not the same as external validation. I suggest that the authors acknowledge this limitation more clearly and avoid overstating generalizability beyond the UK Biobank split used here.

We agree that validation in a fully independent external cohort would provide an important additional assessment of transportability. We have revised the Discussion to make clear that our test set is an internal held-out UK Biobank cohort, not a fully external validation cohort. The present paper is primarily a methods paper introducing and benchmarking the uniLasso framework and its external-score extension, rather than a final PRS release or PRS catalog study. Full external validation of any resulting PRS would require access to external individual-level genotype and phenotype data harmonized to the same endpoints, which is beyond the scope of the present work. We now state this limitation explicitly and identify external validation as an important next step. Please see lines 583-588.

7. Runtime benchmarking should be reported more completely.
Since scalability is an important claim of the paper, please clarify the hardware and threading settings used for each method, whether preprocessing was included consistently across methods, and, if possible, report peak memory usage in addition to wall-clock time.

We thank the reviewer for this helpful suggestion. We have clarified the computational setup and expanded our reporting accordingly. In addition to wall-clock time, we now report peak memory usage for all methods. These results highlight that while uniLasso incurs substantial additional memory due to construction of the LOO matrix, uniLasso-ES avoids this cost and has memory usage comparable to standard Lasso-based approaches. Please see Appendix S2 for our discussion of memory usage.

Minor comments

- Please make the distinction between standard uniLasso and uniLasso ES more prominent throughout the manuscript, especially in the Abstract and Discussion.

We have addressed this in both sections, explaining explicitly that they are different methods which both fit multivariate models using individual-level target data. Specifically, we mention that uniLasso ES uses the external scores to guide (not replace) this fitting. Thank you for pointing this out, we certainly want to ensure the readers are aware of the distinction.

• Claims around sparsity and interpretability should be moderated; selected SNPs in sparse PRS models should not automatically be interpreted as biologically causal variants.

We thank the reviewer for this important clarification and agree that selected variants in sparse models should not be interpreted as biologically causal. We have revised the text to remove causal language.

• For binary traits, if feasible, it would be useful to report or discuss additional evaluation measures beyond AUC, such as calibration or PR-AUC.

Excellent idea! We have added additional evaluation metrics for the binary traits, including both PR-AUC and calibration, which are now reported in Table S2. These metrics show highly similar performance across methods.

• Please make the code/package publicly available.

Thank you for pointing this out. The package is now publicly available on GitHub (link), although it is still under active development. For reproducibility, the folder titled “Examples with Functions Defined” contains the pipeline used prior to constructing the package, and the same functionality is now accessible through the PRS-Lasso package.
