# Supplementary material for: Univariate-guided sparse regression for Biobank-scale high-dimensional omics data
Source: PLoS Genet. 2026 Sep 23;22(9):e1012314. doi: 10.1371/journal.pgen.1012314 (PMC13623159; doi:10.1371/journal.pgen.1012314)
Supplement: Supplementary file 15 [file pgen.1012314.s017.pdf]

***10th August, 2026***

**We thank the editor and Reviewers for very constructive comments and for the opportunity to revise our manuscript. We have now made every effort to incorporate the comments into the revised manuscript. We have addressed the priority items as follows:**

# Reviewers' comments:

## Reviewer #1:

The authors have addressed my comments.

## Reviewer #2:

The revision addressed most of my questions. However, I remain skeptical about highlighting sparsity as a major advantage of uniLasso and equating sparsity with interpretability. This claim is used repeatedly throughout the manuscript in comparisons with other methods, including in a lengthy discussion section. From my perspective, a reduced number of selected variants has neither clear practical nor scientific significance in the context of polygenic prediction. I also do not see how increased sparsity improves interpretation, and the authors do not provide concrete examples or citations to support this point. The assumption that greater sparsity necessarily implies greater interpretability is itself questionable. I would substantially down-weight these claims unless the authors can provide stronger arguments and evidence to support them.

We agree that sparsity alone does not necessarily imply interpretability, and we have revised the manuscript accordingly. We have softened statements linking sparsity to interpretability and instead emphasize the demonstrated reduction in model complexity while maintaining comparable predictive performance. We also note that sparse models *can* provide practical computational advantages in downstream genomic analyses, as illustrated by the recently proposed UltraLasso framework, which leverages sparse active sets for efficient leave-one-chromosome-out (LOCO) prediction within a GPU-accelerated GWAS pipeline. We have included this as a new citation and example within the manuscript (see lines 536-539).

I suggest reporting the incremental R2 or AUC of PRS beyond covariates in all figures and tables.

We agree that this would be relevant information to include. We have added a supplementary figure reporting the incremental predictive performance beyond covariates for all methods. We chose to present these results in the Supplementary Material rather than the main figures and tables to avoid overcrowding. Please see S10 Table.

I'm not sure the statement that PRS-CS "neither incorporates external summary statistics nor induces sparsity in its effect-size estimates" is accurate. Otherwise, by the same logic, most Bayesian methods would not induce sparsity because posterior mean estimates are never exactly zero. In addition, PRS-CS can incorporate additional summary statistics through meta-analysis or through its extensions such as PRS-CSx.

We thank the reviewer for this helpful comment. We have removed the language regarding the incorporation of external summary statistics. We have also clarified that PRS-CS shrinks effect-size estimates *towards* zero but does not induce exact sparsity through feature selection, in contrast to the Lasso-based methods. Please see lines 411- 416.

"PRS were computed and calibrated by fitting a regression model on the validation set, since PRS-CS produces scores that require an estimated scaling coefficient before inclusion in a prediction model." --> I don't think scaling scaling affects AUC or R2, and the authors should use consistent evaluation procedures across PRS methods.

We thank the reviewer for this thoughtful comment. We agree that our original wording was imprecise and could be misinterpreted. We have revised the Methods to clarify that the regression coefficient of the PRS (together with the covariate coefficients) was estimated outside the held-out test set and then held fixed for evaluation. In analyses that included GWAS run in the UKBB, the GWAS was first run in the larger train set and then the model coefficients were fitted in the distinct validation set, to avoid the well known issue of using PRSs in the same sample the GWAS was run in. Thus, the reported R2 and AUC are based on a fixed prediction model rather than on a post hoc rescaling of the PRS. No coefficient or hyperparameter was estimated on the test set. Please see lines 422-430.

"uniLasso does not require explicit LD-matrix modeling" --> This may be technically true, in the sense that uniLasso does not directly compute LD matrices. However, because uniLasso uses individual-level genotype data, correlations among variants are still implicitly modeled.

We thank the reviewer for this very helpful comment. We have revised the manuscript to clarify that, although uniLasso does not require explicit LD-matrix modeling, correlations among variants are nevertheless accounted for implicitly through the individual-level regression model. Please see lines 547-550.

Title "Univariate-guided sparse regression for Biobank-scale high-dimensional omics data" --> Since no other omics data were analyzed, it may be more accurate to refer specifically to genetic data.

We thank the reviewer for this thoughtful suggestion. Although our experiments are conducted using genetic data, the methodological contributions are not specific to genomics and extend naturally to other high-dimensional omics settings. We therefore prefer to retain the current title which reflects the broader applicability of the proposed framework beyond genetic data.

# Reviewer #3:

The revision largely addresses the main concerns. The added Elastic Net, PRS-CS with HapMap3 variants and tuned phi, lassosum2, C+T, uncertainty estimates, and runtime/memory reporting are useful. I do not think further major experiments are needed.

Remaining issues before acceptance:

Some claims are still overstated or inconsistent with the results. For example, “external scores will always boost” is too strong, and uniLasso ES is not consistently sparser than Lasso; for asthma, uniLasso ES selects more variants than Lasso.

We thank the reviewer for pointing this out. We have revised the manuscript to avoid implying that external scores will always improve predictive performance, instead limiting our claims to the phenotypes evaluated in this study. We also corrected the discussion of sparsity to explicitly reflect that uniLasso ES is sparser than Lasso for three of the four phenotypes, but not for asthma.

Please explicitly report incremental predictive performance beyond covariates, e.g. $\Delta R^2$/ΔAUC, at least in the supplement.

We agree that this would be relevant information to include. We have added a supplementary figure reporting the incremental predictive performance beyond covariates for all methods. We chose to present these results in the Supplementary Material rather than the main figures and tables to avoid overcrowding. Please see S10 Table.

Clarify the PRS-CS description. The distinction is not that PRS-CS cannot use external summary statistics, but that it is summary-statistics-based and does not fit an individual-level target-data regression model like uniLasso ES.

Thank you very much for bringing this to our attention. We have revised the manuscript to clarify that, although PRS-CS uses GWAS summary statistics, it does not incorporate them into an individual-level regression model as uniLasso ES does. Please see lines 411-416.

Correct the wording suggesting the test set was used “to obtain optimal coefficients.” The test set should only be used for final evaluation.

We thank the reviewer for catching this imprecise wording. The test set was used exclusively for final evaluation and played no role in model fitting or hyperparameter

selection. We have revised the text to clarify that the model was refit on the combined training and validation sets using the selected regularization parameter, and that the resulting model was then evaluated on the held-out test set. Please see lines 208-212.

Fix minor text issues: “A reviewer raised…” should not appear in the final manuscript; Table 4 says four methods but lists five; “uniLasso EN” appears to be a typo.

Thank you very much for pointing these out. We have corrected each of these issues in the revised manuscript.

Expand data/code availability, especially FinnGen release/access details and a stable code version/commit/DOI.

We thank the reviewer for this important suggestion. We have expanded the Data Availability statement to include the FinnGen release and access details, as well as a stable GitHub release corresponding to the version of the code used for this manuscript.

Moderate claims implying sparse genetic architecture or concentration of SNP heritability; the analyses support sparse prediction, not causal architecture.

This is a very thoughtful comment, thank you. We agree that our analyses support sparse prediction and have revised the manuscript to avoid language that could be interpreted as making claims about the underlying genetic architecture. Accordingly, we have moderated our discussion by removing such language and instead emphasize the empirical finding that substantially sparser models can achieve predictive performance comparable to much denser approaches.
