# Supplementary material for: Univariate-guided sparse regression for Biobank-scale high-dimensional omics data
Source: PLoS Genet. 2026 Sep 23;22(9):e1012314. doi: 10.1371/journal.pgen.1012314 (PMC13623159; doi:10.1371/journal.pgen.1012314)
Supplement: Supplementary file 16 [file pgen.1012314.s018.pdf]

31st August, 2026

We thank the editor and Reviewers for very constructive comments and for the opportunity to revise our manuscript. We have now made every effort to incorporate the comments into the revised manuscript. We have addressed the priority items as follows:

Reviewers' comments:

Reviewer #2:

Some numbers for C+T in the new Table S10 don't make sense. I think they are raw AUC not incremental AUC. Otherwise the authors have addressed my comments.

We thank the reviewer for pointing this out. We have corrected the C+T values in Table S10 to report incremental AUC beyond covariates rather than raw AUC. Please see Table S10.
