# Supplementary material for: Univariate-guided sparse regression for Biobank-scale high-dimensional omics data
Source: PLoS Genet. 2026 Sep 23;22(9):e1012314. doi: 10.1371/journal.pgen.1012314 (PMC13623159; doi:10.1371/journal.pgen.1012314)
Supplement: Peer review history

# Peer review: Decision Letter 0

**DOI:** 10.1371/journal.pgen.1012314.r001

**Authors:** Michael Epstein, Jingjing Yang

30 Apr 2026

PGENETICS-D-26-00283

Univariate-guided sparse regression for Biobank-scale high-dimensional omics data

PLOS Genetics

Dear Dr. Richland,

Thank you for submitting your manuscript to PLOS Genetics. After careful consideration, we feel that it has merit but does not fully meet PLOS Genetics's publication criteria as it currently stands. Therefore, we invite you to submit a revised version of the manuscript that addresses the points raised during the review process.

Please submit your revised manuscript by Jun 28 2026 11:59PM. If you will need more time than this to complete your revisions, please reply to this message or contact the journal office at plosgenetics@plos.org. When you're ready to submit your revision, log on to https://www.editorialmanager.com/pgenetics/ and select the 'Submissions Needing Revision' folder to locate your manuscript file.

Please include the following items when submitting your revised manuscript:

* A letter that responds to each point raised by the editor and reviewer(s). You should upload this letter as a separate file labeled 'Response to Reviewers'. This file does not need to include responses to any formatting updates and technical items listed in the 'Journal Requirements' section below.

* A marked-up copy of your manuscript that highlights changes made to the original version. You should upload this as a separate file labeled 'Revised Manuscript with Track Changes'.

* An unmarked version of your revised paper without tracked changes. You should upload this as a separate file labeled 'Manuscript'.

If you would like to make changes to your financial disclosure, competing interests statement, or data availability statement, please make these updates within the submission form at the time of resubmission. Guidelines for resubmitting your figure files are available below the reviewer comments at the end of this letter.

We look forward to receiving your revised manuscript.

Kind regards,

Jingjing Yang, Ph.D.

Academic Editor

PLOS Genetics

Michael Epstein

Section Editor

PLOS Genetics

Aimée Dudley

Editor-in-Chief

PLOS Genetics

Anne Goriely

Editor-in-Chief

PLOS Genetics

**Journal Requirements:**

1) We ask that a manuscript source file is provided at Revision. Please upload your manuscript file as a .doc, .docx, .rtf or .tex. If you are providing a .tex file, please upload it under the item type u2018LaTeX Source Fileu2019 and leave your .pdf version as the item type u2018Manuscriptu2019.

2) Please upload all main figures as separate Figure files in .tif or .eps format. For more information about how to convert and format your figure files please see our guidelines:

https://journals.plos.org/plosgenetics/s/figures

3) Your current Financial Disclosure states, "The author(s) received no specific funding for this work.".

However, your funding information on the submission form indicates receiving funds. Please ensure that the funders and grant numbers match between the Financial Disclosure field and the Funding Information tab in your submission form. Note that the funders must be provided in the same order in both places as well..

Please amend your detailed Financial Disclosure statement. This is published with the article. It must therefore be completed in full sentences and contain the exact wording you wish to be published.

1) Please clarify all sources of financial support for your study. List the grants, grant numbers, and organizations that funded your study, including funding received from your institution. Please note that suppliers of material support, including research materials, should be recognized in the Acknowledgements section rather than in the Financial Disclosure

2) State the initials, alongside each funding source, of each author to receive each grant. For example: "This work was supported by the National Institutes of Health (####### to AM; ###### to CJ) and the National Science Foundation (###### to AM)."

3) State what role the funders took in the study. If the funders had no role in your study, please state: "The funders had no role in study design, data collection and analysis, decision to publish, or preparation of the manuscript."

4) If any authors received a salary from any of your funders, please state which authors and which funders..

Note: If the reviewer comments include a recommendation to cite specific previously published works, please review and evaluate these publications to determine whether they are relevant and should be cited. There is no requirement to cite these works unless the editor has indicated otherwise.

**Reviewers' comments:**

Reviewer's Responses to Questions

Reviewer #1: This paper presents a method to construct polygenic risk score (PRS) based on uniLasso algorithm. UniLasso is a two-step method that first performs univariate feature screening and then applies Lasso regression on the selected variables to build a sparse multivariate model. Overall, I think the method is solid and reflects the latest developments by the Lasso authors in genomic prediction. Below are my comments.

1. Arguably, one of the central challenges in genomic prediction is handling collinearity among features induced by linkage disequilibrium (LD). While I appreciate the advancement of UniLasso in promoting sign consistency between univariate and multivariate coefficient estimates, I was wondering how uniLasso behaves to infer true genetic effects among SNPs in LD, as univariate regression coefficients can have opposite signs from the true multivariate effects (Y = X\beta + \epsilon, \hat{\beta_j} = (x_j^T x_j)^-1 x_j^T y, E[\hat{\beta_j}] = (x_j^T x_j)^-1 x_j^T X \beta which can have different sign with \beta_j). A small simulation example or discussion could help illustrate this point.

2. What is the performance of elastic net for Fig. 1?

3. The cut-off of minor allele frequency to define common SNPs to build PRS model is typically MAF > 0.01 or MAF > 0.05.

4. The statistical foundation of PRS-CS is horseshore prior (https://academic.oup.com/biomet/article/97/2/465/219397?login=true). Although PRS-CS is Bayesian and not forcing the coefficients to be exactly zero, I think it does encourage sparsity in the coefficients by shrinking most of them towards 0 (Line 387-388). There are also some caveats when comparing with PRS-CS: 1) PRS-CS typically restricts analysis to HapMap3 SNPs, so a fair comparison requires using the same SNP set for both UniLasso and PRS-CS; 2) PRS-CS has a tuning parameter phi that can often be tuned to improve the performance.

5. It may be helpful to discuss whether the authors think it is possible to fit UniLasso entirely based on external summary statistics since 1) the individual level data y and X are not accessible for many traits, 2) the sample size of external summary statistics is often much larger than biobank. Currently the most widely used lasso approach for polygenic prediction is lassosum (https://pubmed.ncbi.nlm.nih.gov/28480976/), which fits lasso based on summary statistics.

6. What is the memory usage of uniLasso on the real data compared with other approaches?

7. I cannot find the link to the package.

Reviewer #2: PGENETICS-D-26-00283

Univariate-guided sparse regression for Biobank-scale high-dimensional omics data

Richland et al.

In this paper, Richland and colleagues adapted the Univariate-Guided Sparse Regression (uniLasso) method to ultra-high-dimensional settings, and demonstrated its application to polygenic prediction.

The authors showed that uniLasso achieves comparable predictive performance to standard Lasso while yielding a sparser solution, and further extended the framework by incorporating external GWAS summary statistics, which improves prediction accuracy.

Overall, the methodological development is sound. However, there is a notable gap between the evaluation framework used in this study and current best practices in statistical genetics, which I detail below.

- Comparison with PRS-CS

The comparison between uniLasso and PRS-CS lacks critical detail regarding variant selection.

The manuscript reports using 1,080,968 variants, but does not specify how these were selected.

This number is much smaller than a typical GWAS on imputed variants (often 8–10 million in UK Biobank) but seems to be larger than the set of directly genotyped variants (~800K in UK Biobank if I recall correctly).

This is critical technical detail because PRS-CS uses ~1.2M HapMap3 variants for PRS construction, most of which are available in well-imputed GWAS.

If the authors used a randomly selected or genotyped set of ~1M variants, many variants may not overlap with the PRS-CS reference panel.

This would significantly reduce the effective number of variants used for PRS-CS (possibly <200K), leading to an unfair comparison.

Please align with standard practice by either using full GWAS summary statistics or restricting analyses to HapMap3 variants.

In addition, the manuscript should provide detailed information on sample and variant QC, case/control definitions, and the number of variants used for PRS construction for each method.

- Sparsity and interpretation

The manuscript emphasizes sparsity as a key advantage of uniLasso. However:

(i) In polygenic prediction, the primary objective is to maximize predictive accuracy rather than interpretability. People usually don't care about sparseness and don't interpret selected variants.

(ii) It is unclear whether reducing the number of nonzero coefficients by uniLasso (e.g., from 30K to 20K variants) meaningfully improves interpretability in practice.

In vast majority of applications, thousands or tens of thousands variants will still be selected.

(iii) The authors didn't interpret selected variants and provided any concrete demonstrations of interpretability gains.

(iv) How does the feature selection in uniLasso compare with simply ranking and selecting lead variants from independent genomic loci, or thresholding Bayesian posterior effect sizes?

(v) The authors should also compare uniLasso with clumping and thresholding, which selects lead variants from each genomic locus and can be as sparse and as 'interpretable' as one would like by varying the p-value threshold.

(vi) One advantage of Bayesian shrinkage methods such as PRS-CS is that the SNP weights are more robust when applied to external datasets.

For example, a large number of PRS weights have been released via PGS Catalog, which allows for replication, application, and reuse of trained PRS model.

If weights concentrate on a small set of variants, and some of the variants are missing from target datasets, the predictive performance may decrease dramatically.

It would be useful to discuss the trade-offs between sparsity and robustness, given that highly sparse models may be sensitive to missing variants in external datasets.

(vii) From table 3, it looks that there is limited overlap of selected variants across Lasso-based methods, which raises concerns about feature stability and interpretability.

- Individual-level data vs. summary statistics

The manuscript frames the requirement for individual-level data as a strength, but this may be viewed as a limitation given the limited accessibility of individual-level data for most large-scale GWAS.

A more balanced discussion is warranted.

When incorporating external summary statistics (e.g., FinnGen), the authors should also benchmark against standard approaches that meta-analyze summary statistics from FinnGen and UKB GWAS and run PRS algorithms on the meta-GWAS summary statistics.

- External validation

All evaluations are conducted within UK Biobank by splitting samples into training, validation, and testing sets.

Assessing performance in an independent external dataset would provide a more rigorous evaluation of generalizability and reduce concerns about overfitting.

- Causal variants

The authors claimed that sparse multivariate methods "concentrate weight on (or near) truly causal variants", but this is not justified by the analyses. I feel this is overstated.

In statistical genetics, there exists a set of dedicated fine-mapping methods and evaluation metrics for causal inference.

The limited overlap of selected features between different types of Lasso methods raised additional concerns about whether selected variants are truly causal.

I think the authors should tune down on this point.

- Evaluation metrics

The authors reported R2 and AUC of the combined model (covariates + PRS) rather than reporting the incremental R2 and AUC of PRS beyond covariates, which obscures the predictive value of PRS.

Please also report appropriate uncertainty estimates (e.g., standard errors or confidence intervals) for all predictions.

- Manuscript writing

The manuscript was written with a strong statistical flavor and in some places not for a genetic audience.

The use of examples from finance in the introduction is not aligned with the scope of PLoS Genetics.

The authors may consider either sending the manuscript to a statistical journal or tailoring the framing to better suit the target audience.

Reviewer #3: This manuscript proposes uniLasso and its extension, uniLasso ES, for sparse polygenic prediction in biobank-scale settings. The idea is interesting, and the method appears potentially useful, particularly because it avoids explicit LD-matrix construction and seems scalable to large individual-level datasets. The inclusion of both quantitative and binary traits is also a strength. That said, I think the manuscript would benefit from clearer methodological positioning, fairer benchmarking, and a more careful discussion of limitations.

Major comments

1. The methodological positioning should be clarified more explicitly.

A central point that should be made much clearer is that this is not a purely summary-statistics-based PRS method. Both uniLasso and uniLasso ES ultimately fit a multivariable model using individual-level target data. In uniLasso ES, external summary statistics are used to guide penalties and/or sign constraints, rather than to replace individual-level fitting.

This distinction matters because comparisons to methods such as PRS-CS are informative, but not fully apples-to-apples. PRS-CS is a summary-statistics-based framework that relies on an LD reference, whereas uniLasso ES is an individual-level regression framework that can additionally leverage external summary information. I recommend that the authors make this distinction much clearer in the Introduction, Methods, and Discussion, so that readers do not misinterpret the method class or its intended use case.

2. The distinction between uniLasso and uniLasso ES should be made much clearer.

Based on the reported results and runtime benchmarks, the strongest practical contribution appears to come from uniLasso ES rather than standard uniLasso. Standard uniLasso seems substantially more computationally expensive, especially for binary traits. The manuscript should therefore distinguish more clearly between the methodological/statistical contribution of standard uniLasso and the practical/computational contribution of uniLasso ES.

3. Comparisons involving uniLasso ES do not appear fully information-matched.

uniLasso ES leverages external GWAS summary statistics, whereas some reported baselines appear to rely primarily on target-cohort information. This makes it difficult to distinguish gains due to the modeling strategy itself from gains due to access to additional external information.

In my view, uniLasso ES should ideally be compared against methods given access to the same external data, such as PRS-CS or lassosum/lassosum2 run on the same external summary statistics, and, if feasible, methods based on meta-analyzed internal plus external summary statistics. Without such information-matched comparisons, the practical advantage of uniLasso ES is difficult to assess cleanly.

4. The manuscript should be positioned more clearly relative to prior hybrid approaches.

The paper would benefit from a more explicit discussion of prior work on combining individual-level data with external GWAS summary statistics, particularly Albiñana et al. (AJHG 2021, Leveraging both individual-level genetic data and GWAS summary statistics increases polygenic prediction), which compared meta-GWAS, SCT, and meta-PRS. At minimum, the authors should clarify how uniLasso ES differs conceptually and methodologically from these earlier hybrid/data-combining approaches.

5. “No explicit LD matrix” is a practical advantage, but the LD-related discussion should be more careful.

The fact that the method does not require explicit LD-matrix modeling is attractive. However, it still relies on marginal effect information, which is itself influenced by LD. The Discussion should therefore acknowledge more explicitly the possible sensitivity of the approach to LD mismatch, ancestry differences, phenotype-definition differences, and covariate-adjustment differences between external and target datasets.

6. The manuscript lacks truly external out-of-sample validation, and generalizability therefore remains unclear.

The train/validation/test split within UK Biobank is reasonable for method development, but it remains an internal held-out evaluation within the same cohort. For PRS methods, an important practical question is whether the model transports well to independent external cohorts. This is especially relevant here because the paper emphasizes practical prediction and the use of external summary information. However, the use of external summary statistics is not the same as external validation. I suggest that the authors acknowledge this limitation more clearly and avoid overstating generalizability beyond the UK Biobank split used here.

7. Runtime benchmarking should be reported more completely.

Since scalability is an important claim of the paper, please clarify the hardware and threading settings used for each method, whether preprocessing was included consistently across methods, and, if possible, report peak memory usage in addition to wall-clock time.

Minor comments

• Please make the distinction between standard uniLasso and uniLasso ES more prominent throughout the manuscript, especially in the Abstract and Discussion.

• Claims around sparsity and interpretability should be moderated; selected SNPs in sparse PRS models should not automatically be interpreted as biologically causal variants.

• For binary traits, if feasible, it would be useful to report or discuss additional evaluation measures beyond AUC, such as calibration or PR-AUC.

• Please make the code/package publicly available.

**********

**Have all data underlying the figures and results presented in the manuscript been provided?**

Large-scale datasets should be made available via a public repository as described in the *PLOS Genetics*  data availability policy, and numerical data that underlies graphs or summary statistics should be provided in spreadsheet form as supporting information.

Reviewer #1: None

Reviewer #2: Yes

Reviewer #3: Yes

**********

PLOS authors have the option to publish the peer review history of their article (what does this mean?). If published, this will include your full peer review and any attached files.

If you choose “no”, your identity will remain anonymous but your review may still be made public.

**Do you want your identity to be public for this peer review?**  For information about this choice, including consent withdrawal, please see our Privacy Policy.

Reviewer #1: No

Reviewer #2: No

Reviewer #3: **Yes:**  Yan Zhang

[NOTE: If reviewer comments were submitted as an attachment file, they will be attached to this email and accessible via the submission site. Please log into your account, locate the manuscript record, and check for the action link "View Attachments". If this link does not appear, there are no attachment files.]

**Figure resubmission:**

**Reproducibility:**

To enhance the reproducibility of your results, we recommend that authors of applicable studies deposit laboratory protocols in protocols.io, where a protocol can be assigned its own identifier (DOI) such that it can be cited independently in the future. Additionally, PLOS ONE offers an option to publish peer-reviewed clinical study protocols. Read more information on sharing protocols at https://plos.org/protocols?utm_medium=editorial-email&utm_source=authorletters&utm_campaign=protocols

# Author comment: Author response to Decision Letter 1

**DOI:** 10.1371/journal.pgen.1012314.r002

8 Jun 2026

Attachment Submitted filename: Response to Reviewers.pdf

# Peer review: Decision Letter 1

**DOI:** 10.1371/journal.pgen.1012314.r003

**Authors:** Michael Epstein, Jingjing Yang

13 Jul 2026

PGENETICS-D-26-00283R1

Univariate-guided sparse regression for Biobank-scale high-dimensional omics data

PLOS Genetics

Dear Dr. Richland,

Thank you for submitting your revised manuscript to PLOS Genetics. While one reviewer was satisfied with this revision, the remaining two reviewers had some minor points they felt still needed to be addressed. Therefore, we invite you to submit a revised version of the manuscript that addresses the points raised during the review process.

Please submit your revised manuscript within by Aug 12 2026 11:59PM. If you will need more time than this to complete your revisions, please reply to this message or contact the journal office at plosgenetics@plos.org. When you're ready to submit your revision, log on to https://www.editorialmanager.com/pgenetics/ and select the 'Submissions Needing Revision' folder to locate your manuscript file.

Please include the following items when submitting your revised manuscript:

* A letter that responds to each point raised by the editor and reviewer(s). You should upload this letter as a separate file labeled 'Response to Reviewers'. This file does not need to include responses to formatting updates and technical items listed in the 'Journal Requirements' section below.

* A marked-up copy of your manuscript that highlights changes made to the original version. You should upload this as a separate file labeled 'Revised Manuscript with Track Changes'.

* An unmarked version of your revised paper without tracked changes. You should upload this as a separate file labeled 'Manuscript'.

If you would like to make changes to your financial disclosure, competing interests statement, or data availability statement, please make these updates within the submission form at the time of resubmission. Guidelines for resubmitting your figure files are available below the reviewer comments at the end of this letter.

As the corresponding author, your ORCID iD is verified in the submission system and will appear in the published article. PLOS supports the use of ORCID, and we encourage all coauthors to register for an ORCID iD and use it as well. Please encourage your coauthors to verify their ORCID iD within the submission system before final acceptance, as unverified ORCID iDs will not appear in the published article. Only the individual author can complete the verification step; PLOS staff cannot verify ORCID iDs on behalf of authors.

We look forward to receiving your revised manuscript.

Kind regards,

Jingjing Yang, Ph.D.

Academic Editor

PLOS Genetics

Michael P. Epstein

Section Editor

PLOS Genetics

Aimée Dudley

Editor-in-Chief

PLOS Genetics

Anne Goriely

Editor-in-Chief

PLOS Genetics

**Additional Editor Comments (if provided):**

Please address remain concerns from Reviewer 2 and Reviewer 3, especially the comments provided by Reviewer 2 about rigorously showing the advantages of the proposed method under specific scenarios.

**Reviewers' comments:**

Reviewer's Responses to Questions

**Comments to the Authors:**

**Please note here if the review is uploaded as an attachment.**

Reviewer #1: The authors have addressed my comments.

Reviewer #2: The revision addressed most of my questions. However, I remain skeptical about highlighting sparsity as a major advantage of uniLasso and equating sparsity with interpretability. This claim is used repeatedly throughout the manuscript in comparisons with other methods, including in a lengthy discussion section. From my perspective, a reduced number of selected variants has neither clear practical nor scientific significance in the context of polygenic prediction. I also do not see how increased sparsity improves interpretation, and the authors do not provide concrete examples or citations to support this point. The assumption that greater sparsity necessarily implies greater interpretability is itself questionable. I would substantially down-weight these claims unless the authors can provide stronger arguments and evidence to support them.

I suggest reporting the incremental R2 or AUC of PRS beyond covariates in all figures and tables.

I'm not sure the statement that PRS-CS "neither incorporates external summary statistics nor induces sparsity in its effect-size estimates" is accurate. Otherwise, by the same logic, most Bayesian methods would not induce sparsity because posterior mean estimates are never exactly zero. In addition, PRS-CS can incorporate additional summary statistics through meta-analysis or through its extensions such as PRS-CSx.

"PRS were computed and calibrated by fitting a regression model on the validation set, since PRS-CS produces scores that require an estimated scaling coefficient before inclusion in a prediction model." --> I don't think scaling scaling affects AUC or R2, and the authors should use consistent evaluation procedures across PRS methods.

"uniLasso does not require explicit LD-matrix modeling" --> This may be technically true, in the sense that uniLasso does not directly compute LD matrices. However, because uniLasso uses individual-level genotype data, correlations among variants are still implicitly modeled.

Title "Univariate-guided sparse regression for Biobank-scale high-dimensional omics data" --> Since no other omics data were analyzed, it may be more accurate to refer specifically to genetic data.

Reviewer #3: The revision largely addresses the main concerns. The added Elastic Net, PRS-CS with HapMap3 variants and tuned phi, lassosum2, C+T, uncertainty estimates, and runtime/memory reporting are useful. I do not think further major experiments are needed.

Remaining issues before acceptance:

Some claims are still overstated or inconsistent with the results. For example, “external scores will always boost” is too strong, and uniLasso ES is not consistently sparser than Lasso; for asthma, uniLasso ES selects more variants than Lasso.

Please explicitly report incremental predictive performance beyond covariates, e.g. ΔR²/ΔAUC, at least in the supplement.

Clarify the PRS-CS description. The distinction is not that PRS-CS cannot use external summary statistics, but that it is summary-statistics-based and does not fit an individual-level target-data regression model like uniLasso ES.

Correct the wording suggesting the test set was used “to obtain optimal coefficients.” The test set should only be used for final evaluation.

Fix minor text issues: “A reviewer raised…” should not appear in the final manuscript; Table 4 says four methods but lists five; “uniLasso EN” appears to be a typo.

Expand data/code availability, especially FinnGen release/access details and a stable code version/commit/DOI.

Moderate claims implying sparse genetic architecture or concentration of SNP heritability; the analyses support sparse prediction, not causal architecture.

**********

**Have all data underlying the figures and results presented in the manuscript been provided?**

Large-scale datasets should be made available via a public repository as described in the *PLOS Genetics*  data availability policy, and numerical data that underlies graphs or summary statistics should be provided in spreadsheet form as supporting information.

Reviewer #1: None

Reviewer #2: Yes

Reviewer #3: None

**********

PLOS authors have the option to publish the peer review history of their article (what does this mean?). If published, this will include your full peer review and any attached files.

If you choose “no”, your identity will remain anonymous but your review may still be made public.

**Do you want your identity to be public for this peer review?**  For information about this choice, including consent withdrawal, please see our Privacy Policy.

Reviewer #1: No

Reviewer #2: No

Reviewer #3: No

[NOTE: If reviewer comments were submitted as an attachment file, they will be attached to this email and accessible via the submission site. Please log into your account, locate the manuscript record, and check for the action link "View Attachments". If this link does not appear, there are no attachment files.]

**Figure resubmission:**

**Reproducibility:**

To enhance the reproducibility of your results, we recommend that authors deposit laboratory protocols in protocols.io, where a protocol can be assigned its own identifier (DOI) such that it can be cited independently in the future. Additionally, PLOS ONE offers an option to publish peer-reviewed clinical study protocols. Read more information on sharing protocols at https://plos.org/protocols?utm_medium=editorial-email&utm_source=authorletters&utm_campaign=protocols

# Author comment: Author response to Decision Letter 2

**DOI:** 10.1371/journal.pgen.1012314.r004

10 Aug 2026

Attachment Submitted filename: Response_to_Reviewers_auresp_2.pdf

# Peer review: Decision Letter 2

**DOI:** 10.1371/journal.pgen.1012314.r005

**Authors:** Michael Epstein, Jingjing Yang

28 Aug 2026

PGENETICS-D-26-00283R2

Univariate-guided sparse regression for Biobank-scale high-dimensional omics data

PLOS Genetics

Dear Dr. Richland,

Thank you for submitting your manuscript to PLOS Genetics. The reviewers were generally satisfied with your revisions. We plan to accept your manuscript once you address one comment from Reviewer 2 noted below. Please submit your revised manuscript within by Sep 27 2026 11:59PM. If you will need more time than this to complete your revisions, please reply to this message or contact the journal office at plosgenetics@plos.org. When you're ready to submit your revision, log on to https://www.editorialmanager.com/pgenetics/ and select the 'Submissions Needing Revision' folder to locate your manuscript file.

Please include the following items when submitting your revised manuscript:

* A letter that responds to each point raised by the editor and reviewer(s). You should upload this letter as a separate file labeled 'Response to Reviewers'. This file does not need to include responses to formatting updates and technical items listed in the 'Journal Requirements' section below.

* A marked-up copy of your manuscript that highlights changes made to the original version. You should upload this as a separate file labeled 'Revised Manuscript with Track Changes'.

* An unmarked version of your revised paper without tracked changes. You should upload this as a separate file labeled 'Manuscript'.

If you would like to make changes to your financial disclosure, competing interests statement, or data availability statement, please make these updates within the submission form at the time of resubmission. Guidelines for resubmitting your figure files are available below the reviewer comments at the end of this letter.

As the corresponding author, your ORCID iD is verified in the submission system and will appear in the published article. PLOS supports the use of ORCID, and we encourage all coauthors to register for an ORCID iD and use it as well. Please encourage your coauthors to verify their ORCID iD within the submission system before final acceptance, as unverified ORCID iDs will not appear in the published article. Only the individual author can complete the verification step; PLOS staff cannot verify ORCID iDs on behalf of authors.

We look forward to receiving your revised manuscript.

Kind regards,

Jingjing Yang, Ph.D.

Academic Editor

PLOS Genetics

Michael Epstein

Section Editor

PLOS Genetics

Aimée Dudley

Editor-in-Chief

PLOS Genetics

Anne Goriely

Editor-in-Chief

PLOS Genetics

**Journal Requirements:**

If the reviewer comments include a recommendation to cite specific previously published works, please review and evaluate these publications to determine whether they are relevant and should be cited. There is no requirement to cite these works unless the editor has indicated otherwise.

1) Your current Financial Disclosure states, "T.H. was supported by NIH grant R01 GM134483. R.T. was supported by NIH grant R01 GM134483 and NSF grant 19DMS1208164. M.A.R. is in part supported by National Human Genome Research Institute (NHGRI) under award R01HG010140, and by the National Institutes of Mental Health (NIMH) under award R01MH124244 both of the National Institutes of Health (NIH). The funders had no role in study design, data collection and analysis, decision to publish, or preparation of the manuscript.".However, your funding information on the submission form indicates "Order of grants" mismatches. Please indicate by return email the full and correct funding information for your study and confirm the order in which funding contributions should appear. Please be sure to indicate whether the funders played any role in the study design, data collection and analysis, decision to publish, or preparation of the manuscript.

**Reviewers' comments:**

Reviewer's Responses to Questions

**Comments to the Authors:**

**Please note here if the review is uploaded as an attachment.**

Reviewer #2: Some numbers for C+T in the new Table S10 don't make sense. I think they are raw AUC not incremental AUC. Otherwise the authors have addressed my comments.

Reviewer #3: None

**********

**Have all data underlying the figures and results presented in the manuscript been provided?**

Large-scale datasets should be made available via a public repository as described in the *PLOS Genetics*  data availability policy, and numerical data that underlies graphs or summary statistics should be provided in spreadsheet form as supporting information.

Reviewer #2: None

Reviewer #3: None

**********

PLOS authors have the option to publish the peer review history of their article (what does this mean?). If published, this will include your full peer review and any attached files.

If you choose “no”, your identity will remain anonymous but your review may still be made public.

**Do you want your identity to be public for this peer review?**  For information about this choice, including consent withdrawal, please see our Privacy Policy.

Reviewer #2: No

Reviewer #3: No

[NOTE: If reviewer comments were submitted as an attachment file, they will be attached to this email and accessible via the submission site. Please log into your account, locate the manuscript record, and check for the action link "View Attachments". If this link does not appear, there are no attachment files.]

**Figure resubmission:**

**Reproducibility:**

To enhance the reproducibility of your results, we recommend that authors deposit laboratory protocols in protocols.io, where a protocol can be assigned its own identifier (DOI) such that it can be cited independently in the future. Additionally, PLOS ONE offers an option to publish peer-reviewed clinical study protocols. Read more information on sharing protocols at https://plos.org/protocols?utm_medium=editorial-email&utm_source=authorletters&utm_campaign=protocols

# Author comment: Author response to Decision Letter 3

**DOI:** 10.1371/journal.pgen.1012314.r006

1 Sep 2026

Attachment Submitted filename: Response_to_Reviewers_auresp_3.pdf

# Editor report: Decision Letter 3

**DOI:** 10.1371/journal.pgen.1012314.r007

**Authors:** Michael Epstein, Jingjing Yang

7 Sep 2026

Dear Dr Richland,

We are pleased to inform you that your manuscript entitled "Univariate-guided sparse regression for Biobank-scale high-dimensional omics data" has been editorially accepted for publication in PLOS Genetics. Congratulations!

Before your submission can be formally accepted and sent to production you will need to complete our formatting changes, which you will receive in a follow up email. Please be aware that it may take several days for you to receive this email; during this time no action is required by you. Please note: the accept date on your published article will reflect the date of this provisional acceptance, but your manuscript will not be scheduled for publication until the required changes have been made.

Once your paper is formally accepted, an uncorrected proof of your manuscript will be published online ahead of the final version, unless you’ve already opted out via the online submission form. If, for any reason, you do not want an earlier version of your manuscript published online or are unsure if you have already indicated as such, please let the journal staff know immediately at plosgenetics@plos.org.

In the meantime, please log into Editorial Manager at https://www.editorialmanager.com/pgenetics/, click the "Update My Information" link at the top of the page, and update your user information to ensure an efficient production and billing process. Note that PLOS requires an ORCID iD for all corresponding authors. Therefore, please ensure that you have an ORCID iD and that it is validated in Editorial Manager. To do this, go to ‘Update my Information’ (in the upper left-hand corner of the main menu), and click on the Fetch/Validate link next to the ORCID field.  This will take you to the ORCID site and allow you to create a new iD or authenticate a pre-existing iD in Editorial Manager.

If you have a press-related query, or would like to know about making your underlying data available (as you will be aware, this is required for publication), please see the end of this email. If your institution or institutions have a press office, please notify them about your upcoming article at this point, to enable them to help maximise its impact. Inform journal staff as soon as possible if you are preparing a press release for your article and need a publication date.

Thank you again for supporting open-access publishing; we are looking forward to publishing your work in PLOS Genetics!

Yours sincerely,

Jingjing Yang, Ph.D.

Academic Editor

PLOS Genetics

Michael Epstein

Section Editor

PLOS Genetics

Aimée Dudley

Editor-in-Chief

PLOS Genetics

Anne Goriely

Editor-in-Chief

PLOS Genetics

www.plosgenetics.org

BlueSky: @plos.bsky.social

----------------------------------------------------

Comments from the reviewers (if applicable):

----------------------------------------------------

**Data Deposition**

If you have submitted a Research Article or Front Matter that has associated data that are not suitable for deposition in a subject-specific public repository (such as GenBank or ArrayExpress), one way to make that data available is to deposit it in the Dryad Digital Repository. As you may recall, we ask all authors to agree to make data available; this is one way to achieve that. A full list of recommended repositories can be found on our website.

The following link will take you to the Dryad record for your article, so you won't have to re‐enter its bibliographic information, and can upload your files directly:

http://datadryad.org/submit?journalID=pgenetics&manu=PGENETICS-D-26-00283R3

More information about depositing data in Dryad is available at http://www.datadryad.org/depositing. If you experience any difficulties in submitting your data, please contact help@datadryad.org for support.

Additionally, please be aware that our data availability policy requires that all numerical data underlying display items are included with the submission, and you will need to provide this before we can formally accept your manuscript, if not already present.

----------------------------------------------------

**Press Queries**

If you or your institution will be preparing press materials for this manuscript, or if you need to know your paper's publication date for media purposes, please inform the journal staff as soon as possible so that your submission can be scheduled accordingly. Your manuscript will remain under a strict press embargo until the publication date and time. This means an early version of your manuscript will not be published ahead of your final version. PLOS Genetics may also choose to issue a press release for your article. If there's anything the journal should know or you'd like more information, please get in touch via plosgenetics@plos.org.

# Editor report: Acceptance letter

**DOI:** 10.1371/journal.pgen.1012314.r008

**Authors:** Michael Epstein, Jingjing Yang

PGENETICS-D-26-00283R3

Univariate-guided sparse regression for Biobank-scale high-dimensional omics data

Dear Dr Richland,

We are pleased to inform you that your manuscript entitled "Univariate-guided sparse regression for Biobank-scale high-dimensional omics data" has been formally accepted for publication in PLOS Genetics! Your manuscript is now with our production department and you will be notified of the publication date in due course.

The corresponding author will soon be receiving a typeset proof for review, to ensure errors have not been introduced during production. Please review the PDF proof of your manuscript carefully, as this is the last chance to correct any errors. Please note that major changes, or those which affect the scientific understanding of the work, will likely cause delays to the publication date of your manuscript.

Soon after your final files are uploaded, unless you have opted out or your manuscript is a front-matter piece, the early version of your manuscript will be published online. The date of the early version will be your article's publication date. The final article will be published to the same URL, and all versions of the paper will be accessible to readers.

For Research Articles, you will receive an invoice from PLOS for your publication fee after your manuscript has reached the completed accept phase. If you receive an email requesting payment before acceptance or for any other service, this may be a phishing scheme. Learn how to identify phishing emails and protect your accounts at https://explore.plos.org/phishing.

Thank you again for supporting PLOS Genetics and open-access publishing. We are looking forward to publishing your work!

With kind regards,

Sharmila Kamatchi

PLOS Genetics

On behalf of:

The PLOS Genetics Team

Carlyle House, Carlyle Road, Cambridge CB4 3DN | United Kingdom

plosgenetics@plos.org | +44 (0) 1223-442823

plosgenetics.org | Twitter: @PLOSGenetics
